# Genome-Driven Discovery of Enzymes with Industrial Implications from the Genus *Aneurinibacillus*

**DOI:** 10.3390/microorganisms9030499

**Published:** 2021-02-26

**Authors:** Majid Rasool Kamli, Nada A. Y. Alzahrani, Nahid H. Hajrah, Jamal S. M. Sabir, Adeel Malik

**Affiliations:** 1 Department of Biological Sciences, Faculty of Science, King Abdulaziz University, Jeddah 21589, Saudi Arabia; nhajrah260@gmail.com (N.H.H.); jsabir2622@gmail.com (J.S.M.S.); 2 Center of excellence in Bionanoscience Research, King Abdulaziz University, Jeddah 21589, Saudi Arabia; nayalzahrani@kau.edu.sa; 3 Institute of Intelligence Informatics Technology, Sangmyung University, Seoul 30316, Korea

**Keywords:** *Aneurinibacillus*, pan-genome, carbohydrate-active enzymes (CAZymes), biosynthetic gene clusters, heavy metal resistance

## Abstract

Bacteria belonging to the genus *Aneurinibacillus* within the family *Paenibacillaceae* are Gram-positive, endospore-forming, and rod-shaped bacteria inhabiting diverse environments. Currently, there are eight validly described species of *Aneurinibacillus*; however, several unclassified species have also been reported. *Aneurinibacillus* spp. have shown the potential for producing secondary metabolites (SMs) and demonstrated diverse types of enzyme activities. These features make them promising candidates with industrial implications. At present, genomes of 9 unique species from the genus *Aneurinibacillus* are available, which can be utilized to decipher invaluable information on their biosynthetic potential as well as enzyme activities. In this work, we performed the comparative genome analyses of nine *Aneurinibacillus* species representing the first such comprehensive study of this genus at the genome level. We focused on discovering the biosynthetic, biodegradation, and heavy metal resistance potential of this under-investigated genus. The results indicate that the genomes of *Aneurinibacillus* contain SM-producing regions with diverse bioactivities, including antimicrobial and antiviral activities. Several carbohydrate-active enzymes (CAZymes) and genes involved in heavy metal resistance were also identified. Additionally, a broad range of enzyme classes were also identified in the *Aneurinibacillus* pan-genomes, making this group of bacteria potential candidates for future investigations with industrial applications.

## 1. Introduction

The family *Paenibacillaceae* represents a diverse group of bacteria that belongs to the phylum Firmicutes [1]. Currently, this bacterial family consists of 16 genera, namely: *Paenibacillus*, *Ammoniibacillus*, *Ammoniphilus*, *Aneurinibacillus*, *Brevibacillus*, *Chengkuizengella*, *Cohnella*, *Fontibacillus*, *Gorillibacterium*, *Longirhabdus*, *Marinicrinis*, *Oxalophagus*, *Paludirhabdus*, *Saccharibacillus*, *Thermobacillus*, and *Xylanibacillus* [2]. Most of the bacteria in this family are considered to have significant environmental functions and can be used as potential biotechnological candidates. Among the *Paenibacillaceae* family, the genus *Paenibacillus* has been widely studied. The isolated strains have been investigated for their role in promoting plant growth, nutrient cycling, production of exopolysaccharides (EPS), and production of secondary metabolites [3]. Furthermore, many *Paenibacillus* species may be used in insect pest control and can produce antimicrobial compounds [4]. *Brevibacillus* is another important genus of this family. Members of this genus produce promising secondary metabolites with antimicrobial activity such as tauramamide, which can inhibit *Enterococcus* spp. [5] The genus *Aneurinibacillus* was suggested as a new genera of bacteria based on the reclassification of the *Bacillus aneurinolyticus* and related members of the *Bacillus* genus [6]. Bacteria belonging to the genus *Aneurinibacillus* are Gram-positive, endospore-forming, and rod-shaped bacteria, and have been reported from diverse habitats [6,7,8,9,10,11,12,13]. At the time of writing, the list of prokaryotic names with standing in nomenclature (LPSN) [14] database comprises eight validly named species of the genus *Aneurinibacillus.* An additional species named “*Aneurinibacillus humi*” [7] is also described but not yet published validly. In addition to these nine species, many unclassified *Aneurinibacillus* strains have been reported [15].

Members of the genus *Aneurinibacillus* have shown many essential functions with industrial implications. For example, they can act as promising biosurfactants, produce secondary metabolites, possess biocontrol abilities, and exhibit potential environmental applications [16,17,18,19]. One of the essential applications of *Aneurinibacillus* is its biocontrol potential. Studies have reported their role in controlling plant diseases’ development by producing cyclic peptide GS (gramicidin S) [19,20]. Their biocontrol potential differs among different isolated strains, and strains belonging to the same species showed varying biological control abilities [16]. Among the *Aneurinibacillus* bacteria, *A. aneurinilyticus* and *A. migulanus* are two well-known species with plant growth-promoting properties and antimicrobial cyclic peptide gramicidin-forming capacities [16,21]. Recently, it was reported that *Aneurinibacillus* sp. H1 has potential for the biosynthesis of polyhydroxyalkanoates (PHA) on an industrial scale, and its capacity for production of copolymers such as 3-hydroxybutyrate (3HB), 4-hydroxybutyrate (4HB), and 3-hydroxyvalerate (3HV) with exceptionally high 4HB and 3HV fractions [22]. Furthermore, *A. aneurinilyticus* DBT87 isolated from the swine and goat can produce cellulase and xylanase enzymes, which can degrade lignocellulose biomass [23]. Many *Aneurinibacillus* strains have also shown promising potential for multiple heavy metal resistance [24,25]. These data suggest that the genus *Aneurinibacillus* represents an extremely promising candidate with a broad range of biotechnological applications.

With advances in genome sequencing technology, it has been speculated that microorganisms remain an important source for discovering novel biosynthetic compounds [26]. Whole-genome sequencing provides a promising technique for mining the biosynthetic potential as well as the discovery of enzymes with industrial implications [27,28,29]. At present, about 20 *Aneurinibacillus* genomes are available in the PATRIC [30] database. Among these, only nine genomes represent the unique species, making this genus one of the least studied genera within the family *Paenibacillaceae*, especially at the genomic level. Most of the genomic-based studies for the genus *Aneurinibacillus* are just genome announcements with limited scope for analyses [20,31,32,33,34,35]. Others were focused on deciphering the secondary metabolite biosynthesis potential of the genus *Aneurinibacillus* [36]. For example, genome data of three well-studied *Aneurinibacillus* strains (*A. migulanus*, *A. terranovensis*, and *A. aneurinilyticus*) revealed the existence of secondary metabolite (SM)-producing biosyntthetic gene clusters (BGCs), thus displaying their ability for the production of new bioactive compounds [36]. It was shown that these clusters have the potential for producing a broad range of secondary metabolites such as bacteriocins, microcins, non-ribosomal peptides, polyketides, terpenes, phosphonates, lasso peptides, and linaridins. However, only a limited number of genomes were used in such studies. This limits our understanding of the complete potential of microorganisms, which have shown great capabilities for diverse types of enzyme activities, or the production of bioactive compounds [16,22,37,38,39,40]. Therefore, in this work we apply genome mining approaches to investigate the biosynthetic potential as well as explore the diverse sets of enzymes for biomass degradation and heavy metal resistance present in *Aneurinibacillus* genomes. Briefly, we observe that in addition to the presence of some common enzyme classes in the *Aneurinibacillus* pan-genome, specific enzyme categories were also found in each component (core, accessory, and unique) of this pan-genome. We also identified several enzymes which may have biotechnological implications in biodegradation, secondary metabolite biosynthesis, and heavy metal resistance. The current work represents the first comprehensive comparative analysis of the genus *Aneurinibacillus* by using the publicly available genomes of this underrepresented bacterial group. The findings of the current study might help us further discern the biosynthetic potential and discover a broad set of enzymes that may have industrial significance.

## 2. Materials and Methods

### 2.1. Datasets

The PATRIC [30] database was searched for the available *Aneurinibacillus* genomes, which resulted in 20 genomes with a varying number of contigs. In species with multiple strains, only 1 representative strain was selected if it was a type strain or consisted of a minimum number of contigs. For example, there were 8 genomes available for *A. migulanus*, out of which only one was selected for comparative analysis. Therefore, nine *Aneurinibacillus* genomes were selected for further analysis (Table 1). The GenBank, genome, and proteome files for each of these representative strains were downloaded from the NCBI genome [41] or PATRIC [30] databases, respectively. The completeness of nine *Aneurinibacillus* genomes were assessed by searching for the presence of 450 core bacillales genes (bacillales_odb10 lineage) using BUSCO v4.0.6 [42].

### 2.2. Genome Annotation

Regions containing possible BGCs with SM-producing potential were predicted by antiSMASH v5.1.0 [43] with default parameters. Enzyme commission (EC) numbers were predicted for all CDS using DeepEC [44] tool. In contrast, putative carbohydrate-active enzymes (CAZymes) [45] were assigned with dbCAN2 meta server [46] with an e-value cut-off of ≤10^–5^. A BLASTP [47] based search against the BacMet v2.0 database [48] was performed to identify genes involved in antibacterial and heavy metal resistance, respectively.

### 2.3. Phylogenomic and Comparative Genome Analyses

Genome-based taxonomic analysis was performed by using the Type (Strain) Genome Server (TYGS) [49], and the tree visualized with MEGA X software [50]. Moreover, the pan-genome analysis was performed using the Bacterial Pan Genome Analysis (BPGA v1.3) [51] and bacterial pan-genome profile (PanGP v1.0.1) [52] tools at a threshold of 40% sequence identity.

## 3. Results and Discussion

### 3.1. General Features of Aneurinibacillus Genome

Currently, there are eight validly named *Aneurinibacillus* species in the LPSN [14] database. Almost all these strains have their genomes available in the databases except for *A. sediminis*. Moreover, the genome belonging to two additional strains namely, *Aneurinibacillus* sp. UBA3580 (PRJNA348753) and *Aneurinibacillus* sp. XH2 (PRJNA287204) are also available. Therefore, the genome sequences of these nine *Aneurinibacillus* species were downloaded from the NCBI genome database based on the criteria described in the “Methods” section. The number of contigs for these genomes ranged between 1 (*A. soli* CB4^T^) and 395 (*A. aneurinilyticus* ATCC 12856^T^). Among all, only the genomes of *A. soli* CB4^T^ and *Aneurinibacillus* sp. XH2 were complete, whereas the remaining represented draft genomes. The number of contigs for these draft genomes ranged between 28 (*A. migulanus* DSM 2895^T^) and 395 (*A. aneurinilyticus* ATCC 12856^T^). The *Aneurinibacillus* genomes exhibited great diversity in size, with an average length of 4.56 Mb (± 1.0). A difference of about 3Mb in size was observed between the strains with the largest (*A. migulanus* DSM 2895^T^) and smallest (*Aneurinibacillus* sp. UBA3580) genomes. Table 1 summarizes the genomic features such as CDS, GC content, isolation source, etc., for all nine *Aneurinibacillus* strains analyzed in this work. BUSCO analysis of these *Aneurinibacillus* genomes suggests the presence of a very complete set of core genes from the order *bacillales* with an overall completeness ranging between 91.5 to 99.5% (Table 1). Moreover, the level of duplication, fragmentation and number of missing genes was very low (Table S1). These high genome completeness scores imply that the datasets are reliable for any subsequent analysis.

### 3.2. Phylogenomic Analysis

**Figure 1:**
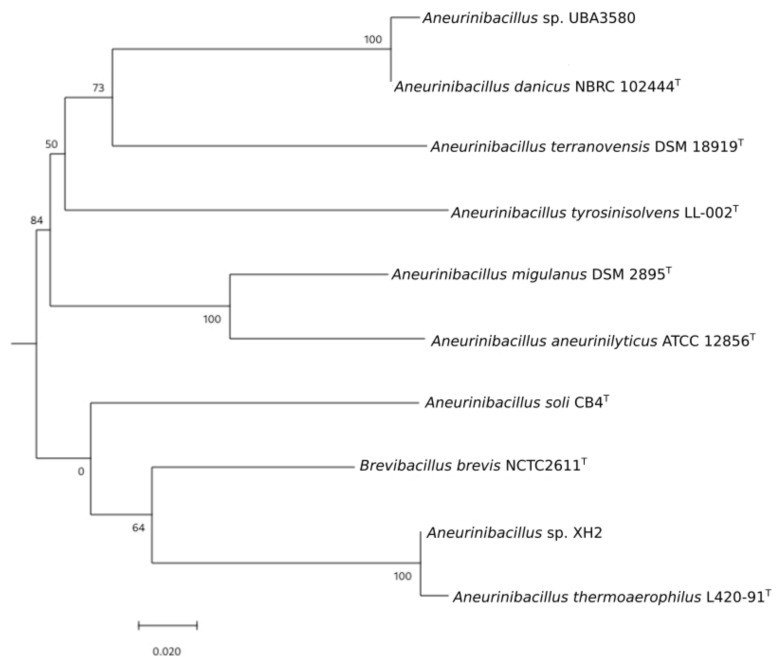
Phylogenomic tree of all *Aneurinibacillus* strains used in this study. The branch lengths are scaled in terms of GBDP distance formula *d5*. The numbers above branches are GBDP pseudo-bootstrap support values >60% from 100 replications, with an average branch support of 81.6%. The genome of *Brevibacillus brevis* NCTC 2611^T^ was used as an out-group.

The genome sequences of all *Aneurinibacillus* strains were utilized as a phylogenetic marker to check the taxonomic grouping of the genus *Aneurinibacillus*. These strains were divided into several subgroups when the genomes of all the strains were used to generate the phylogenomic tree (Figure 1). Specifically, strains that show similar genomic properties in terms of genome size and GC content were clustered together. For example, *A. aneurinilyticus* ATCC 12856^T^ and *A. migulanus* DSM 2895^T^ are clustered together and have large genomes (5.30–6.35Mb). These two strains also showed the lowest average GC content of 43.07 (±0.18) compared to other *Aneurinibacillus* strains. The dDDH score between these two strains was <70%, clearly distinguishing them as distinct species (Table 2).

Similarly, strains with the smallest genomes namely, *Aneurinibacillus* sp. XH2 and *A. thermoaerophilus* L 420-91^T^ were grouped on the phylogenomic tree. The dDDH score between these two species was >70%. The other two strains exhibiting such high dDDH scores of >70% between them were *A. danicus* NBRC 102444^T^ and *Aneurinibacillus* sp. UBA3580 (Table 2). These values are very much above the 70% threshold of species delineation [53]. Additional taxonomic-based studies would elucidate the precise taxonomic association of *Aneurinibacillus sp*. UBA3580, and *Aneurinibacillus* sp. XH2 within the genus *Aneurinibacillus* as more genomes from this bacterial group become available.

### 3.3. Abundant Enzyme Classes of the Aneurinibacillus Pan-Genome

**Figure 2:**
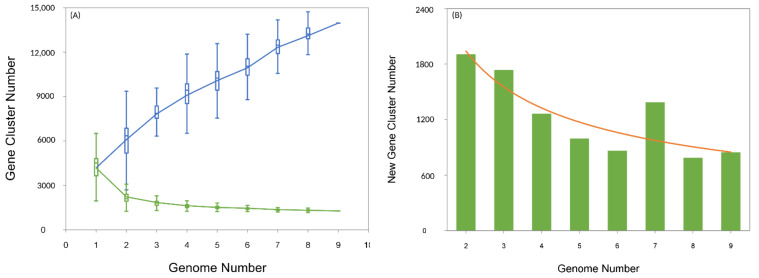
Pan-genome analysis of nine *Aneurinibacillus* species. (**A**) Core (green) vs. pan (blue) developmental plot. (**B**) Relationship between new genes and serial addition of sequenced genomes.

The pan-genome analysis of nine *Aneurinibacillus* genomes suggested that 1265 sequences were highly conserved in all strains and represented the core genome. Sequences belonging to the core genome perform indispensable basic cellular functions, including survival, and bestow the key phenotypic traits. The accessory/dispensable genome comprises 18,608 sequences and ranged between 1353 (*A. terranovensis* DSM 18919^T^) and 2850 (*A. migulanus* DSM 2895^T^) genes. Broadly, the accessory genome components are responsible for diversity within a species and may accomplish roles that are trivial for bacterial growth but might help overcome antagonistic environmental surroundings [54]. Similarly, the number of strain-specific (unique) genes was also highly variable, and on average *Aneurinibacillus* strains consisted of at least 845.44 (± 573.63) unique genes. Individually, the genomes of *Aneurinibacillus* sp. XH2 and *A. tyrosinisolvens* LL-002^T^ contained 88 and 1725 unique genes, respectively. The *Aneurinibacillus* pan-genome exhibits the features of an “open” pan-genome [54], the size of which increases with the subsequent inclusion of new genomes (Figure 2A). As expected, the core genome displays a steady decline in size as new genomes are added. On the other hand, the number of new genes does not reach 0 with further addition of new genomes (Figure 2B). These conclusions are based on the power-law regression analysis, which illustrates an “open” pan-genome for the genus *Aneurinibacillus* with *Bpan* = 0.53. Such pan-genomes are generally observed in bacterial species from various environments with intricate lifestyles and tend towards horizontal gene transfer (HGT) [54,55]. However, it should also be noted that the open pan-genome reported in this study is based on a limited number of available *Aneurinibacillus* genomes. The core, accessory, and unique genome sequences are available from [56].

Besides their roles in metabolic processes, enzymes of microbial origin are widely used for different industrial processes. Therefore, to get a general overview of the types of various enzymes present in this genus, we explored the major and specific enzyme classes present in the *Aneurinibacillus* pan-genome and highlight their potential industrial implications.

**Figure 3:**
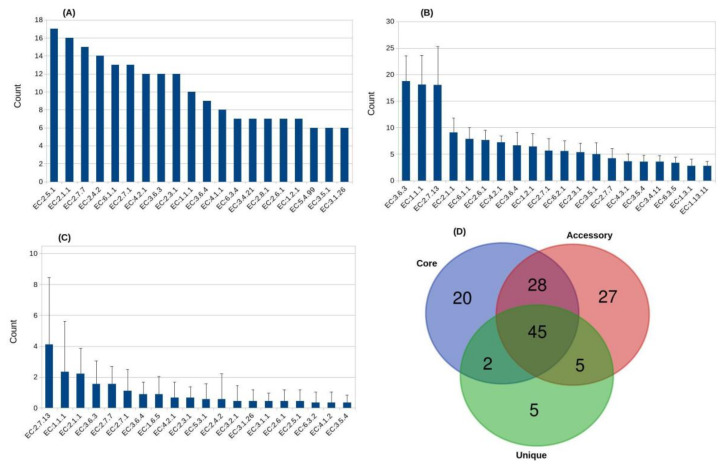
Distribution of the top-most enzyme classification (EC) numbers in the *Aneurinibacillus* pan-genome; core (**A**), accessory (**B**), and unique (**C**) genomes. Venn diagram showing the number of common and pan-genome-specific EC classes (**D**).

Almost 30% of the core genome sequences were assigned to 95 various enzyme sub-classes. Among them, the top 3 represent multiple types of transferases, including enzymes that transfer alkyl or aryl groups, except methyl groups (EC:2.5.1), methyltransferases (EC:2.1.1), and nucleotidyltransferases (EC:2.7.7) (Figure 3A). Enzymes belonging to the methyltransferase (MT) family represent a diverse set of proteins that are involved in a broad range of cellular functions such as regulation of gene expression, biosynthesis, and signaling [57]. MTs such as carboxyl MTs (CMTs) methylate the hydroxyl oxygen of carboxylic acids to form a methyl ester [58]. The core genome of *Aneurinibacillus* contained a CMT enzyme (EC: 2.1.1.80), suggesting that this enzyme was found in each organism and may be involved in carrying out basic functions necessary for survival. However, an additional CMT enzyme was found in the species-specific genome of *A. terranovensis*. Recently, it has been reported that in addition to their known biological roles, MTs such as CMTs have potential applications in pharmaceuticals, biofuels, and bioplastics [58]. Overall, 20 enzyme sub-classes were specific to this core genome of *Aneurinibacillus*, suggesting their essential house-keeping roles (Table S2).

A significant percentage (10.47%) of the accessory genome representing 1949 sequences were assigned to 105 different enzyme sub-classes. The most abundant enzyme sub-classes of the accessory genome are shown in Figure 3B and categories specific to the accessory genome are summarized in Table S2. Among these, enzymes belonging to the group EC:3.6 are involved in the hydrolysis of acid anhydrides [59], and several sub-subclasses belonging to this group are now transferred to various classes of translocases (EC:7). Oxidoreductases (EC:1) in bacteria play an essential role in detoxifying toxic organic compounds through oxidative coupling [60]. They act on the CH-OH group of donors with NAD(+) or NADP(+) as an acceptor and can be either oxidases or dehydrogenases (EC:1.1.1). From the biotechnological point of view, many biocatalytic-based applications of oxidoreductases have long been an important objective. For example, developing diagnostic tests, improving biosensors, constructing novel systems for regeneration of indispensable coenzymes, developing bioreactors for biodegradation of pollutants, and designing oxidoreductase-based approaches for synthesis of polymers and oxyfunctionalized organic substrates [61]. One of the essential enzymes among dehydrogenases is alcohol dehydrogenases (EC:1.1.1.1), which catalyzes the interconversion between alcohols and aldehydes or ketones [62]. At least 11 representatives of EC.1.1.1.1 were found exclusively in the accessory genome. These alcohol dehydrogenases are promising candidates for biotechnological applications especially in pharmaceutical industry [62]. Enzymes belonging to sub-class EC:2.7.13 are histidine kinases (Hks), which show autophosphorylase, transphosphorylase, and dephosphorylase activities [63]. Many bacterial and fungal histidine kinases are promising drug targets [64,65,66]. Their antimicrobial, antifungal resistance, and virulence regulation makes them potential enzymes for industrial applications. The number of sequences assigned to various enzyme sub-classes ranged between 218 (*A. migulanus* DSM 2895) and 170 (*Aneurinibacillus* sp. UBA3580) in individual accessory genomes. Another enzyme of the accessory genome that was identified in only three (*A. soli*, *A. terranovensis*, and *A. tyrosinisolvens*) genomes was selenophosphate synthase (SPS; EC: 2.7.9.3), also known as selenide, water dikinase (Figure S1). Of these only SPS from *A. soli* was predicted by DeepEC, whereas the other two were identified after manual inspection of all *Aneurinibacillus* proteomes. The SPS enzyme catalyzes the synthesis of selenium donor selenophosphate, which is required for the biosynthesis of selenocysteine and 2-selenouridine residues in seleno-tRNA [67]. The selenocysteine is in turn integrated into proteins during translation to form selenoproteins that are essential to various cellular processes. Similarly, selenocysteine synthase (EC:2.9.1.1), an enzyme also involved in the biosynthesis of selenocysteine, was found only in the accessory genomes of *A. terranovensis*, and *A. tyrosinisolvens* [68]. Selenocysteine (the 21st amino acid) is unique among all other amino acids as it contains an indispensable dietary micronutrient (selenium). It is also the only amino acid being encoded by the codon UGA, and represents the sole amino acid synthesized on its tRNA across all the domains of life [69]. In contrast, the accessory genomes of *A. migalunus*, *A. thermoaerophilus*, and *Aneurinibacillus* sp. XH2 contained phosphonoacetylaldehyde phosphonohydrolase, also known as phosphonatase (EC: 3.11.1.1). Specifically, phosphonatase triggers the hydrolysis of C-P bond of phosphonoacetaldehyde into acetaldehyde and orthophosphate [70]. This is the second reaction in a two-step chemical pathway used by many bacteria to degrade the ubiquitous natural phosphonate 2-aminoethylphosphonate (AEP) into useable forms of nitrogen, carbon, and phosphorus [71]. Enzymes capable of breaking such C-P bonds are known to play an essential role in natural detoxification because many herbicides, insecticides, and flame retardants belong to this organophosphate group of compounds [72].

One of the important enzymes of the isoprenoid biosynthetic pathway known as isopentenyl-diphosphate delta-isomerase (EC:5.3.3.2; IDI) [73] was found in the accessory genomes of all *Aneurinibacillus* strains except *A. soli*. This enzyme (IDI) triggers the interconversion of isopentenyl diphosphate (IPP) and dimethylallyl diphosphate (DMAPP), a crucial step through which mevalonate enters into the isoprenoid biosynthetic pathway. Isoprenoids, also known as terpenoids, are essential components of all living organisms, and have been implicated in pharmaceutical, flavor fragrance, and biofuel industries [74].

Of the total 7609 singletons, 242 (3.18%) sequences were assigned to 57 enzyme sub-classes. This number is minimal compared to the percentage of various enzymes detected in the core and accessory genomes. Although the distribution of most abundant enzyme sub-classes was more or less similar to the accessory genome, the top-most enzyme type to be observed in the species-specific genome was EC:2.7.13 (with 37 sequences) as compared to EC:3.6.3 and EC:2.5.1 in the accessory and core genomes, respectively (Figure 3C). The other two top enzyme sub-classes in the unique genome included EC:1.1.1 and methyltransferases (EC:2.1.1), with 21 and 20 members assigned to these groups. Overall, the distribution of these 242 sequences exhibited a significant variation. For example, 62 out of 1049 unique sequences from *A. terranovensis* DSM 18919 were assigned to 29 enzyme sub-classes. In contrast, only two singletons (out of 88) from *Aneurinibacillus* sp. XH2 represented two different enzyme groups.

Overall, at least 45 sub-classes were commonly found in all the pan-genome components (i.e., core, accessory, and unique genomes). Twenty sub-classes are specific to the core genome and were not identified in other two pan-genome components. In contrast, 27 enzyme sub-classes were found to be specific for the accessory genome. A minimal number of only five sub-classes were particular to the unique genome (Figure 3D and Table S2), and included categories such as with reduced pteridine as one donor (EC:1.14.13), with dinitrogen as acceptor (EC:1.18.6), other oxidoreductases (EC:1.97.1), exoribonucleases producing 3’-phosphomonoesters (EC:3.1.4), and other intramolecular oxidoreductases (EC:5.3.99), respectively. Among these, three nitrogenases (EC:1.18.6.1) were found only in the strain-specific genome of *A. terranovensis*. Two of these nitrogenase genes encode alpha and beta subunits of nitrogenase molybdenum-iron protein, whereas the third encodes a nitrogenase iron protein. Three additional nitrogenases representing nitrogenase iron-molybdenum cofactor biosynthesis protein (NifE), nitrogenase iron-molybdenum cofactor biosynthesis protein (NifN), and nitrogenase cofactor biosynthesis protein (NifB) were specifically identified in the genome of *A. terranovensis.* However, these three enzymes were not assigned to any class by DeepEC tool. No such nitrogenase enzyme was identified in other *Aneurinibacillus* genomes. Microorganisms use such nitrogenases for converting atmospheric N_2_ to ammonia, an indispensable source of N atoms for higher organisms [75]. Similarly, a pyruvate formate-lyase]-activating enzyme (PFL-AE), also known as PFL activase (EC:1.97.1.4) was only identified in the genome of this *Aneurinibacillus* strain. PFL-AE is one of the first-discovered members of the “AdoMet radical” or “radical SAM” superfamily of enzymes which act on a broad array of biomolecules in many pathways [76]. Although other *Aneurinibacillus* strains did have radical SAM proteins in their genomes, none of these exhibited statistically significant sequence similarity with the PFL-AE from *A. terranovensis.*

Additionally, an oxidoreductase representing dimethylsulfide (DMS) monooxygenase (EC:1.14.13.131) was found only in the genome of *A. danicus*. In some bacteria DMS monooxygenase is involved in the first step of DMS degradation pathways. DMS is a volatile organosulfur compound essential for the biogeochemical cycling of sulfur and the regulation of global climate [77]. This enzyme (EC:1.14.13.131) from *A. danicus* exhibited ~54% sequence identity with the known DMS monooxygenease from *Hyphomicrobium sulfonivorans* [77]. Another oxidoreductase that was found only in the unique genome of *A*. *tyrosinisolvens* was inosose isomerase (EC:5.3.99.11), also known as 2-keto-myo-inositol isomerase (KMI isomerase), which converts 2KMI to 1-keto-d-*chiro*-inositol (1KDCI), a precursor for the synthesis of d-*chiro*-Inositol (DCI). The reduction of 1KDCI to DCI is triggered by the action of inositol 2-dehydrogenase (also found in *A. tyrosinisolvens*). DCI is a promising drug candidate for the treatment of type 2 diabetes and polycystic ovary syndrome [78].

Examples of nucleoside (both purine and pyrimidine) phosphorylase enzymes representing EC:2.4.2.1 and EC:2.4.2.2 were also found in the pan-genome of Aneurinibacillus strains. Recently, a new thermostable trimeric purine nucleoside phosphorylase was cloned and characterized from Aneurinibacillus migulanus AM007 [38]. Such enzymes are used as biocatalysts for the synthesis of pentose-1-phosphates and nucleoside analogues that represent an important source of many drugs including antiviral and anticancer drugs [79].

### 3.4. Aneurinibacillus Enzymes with Significant Industrial Implications

Recognizing their importance in a broad range of industrial applications, an attempt has been made to highlight the significance of different enzymes focusing on secondary metabolite-producing BGCs, CAZymes, and heavy metal resistance.

#### 3.4.1. Biosynthetic Potential of *Aneurinibacillus*

To explore the biosynthetic potential of *Aneurinibacillus* strains, we used antiSMASH to further identify the potential regions that may participate in the production of biosynthetic compounds. Overall, 60 such regions with biosynthetic potential were identified in all *Aneurinibacillus* genomes, and their number in each strain ranged between 2 (*A. terranovensis* DSM 18919) and 10 (*A. aneurinilyticus* ATCC 12856^T^ and *A. migulanus*). Moreover, no commonly occurring or core biosynthetic cluster was identified in *Aneurinibacillus* genomes. Of the 60 biosynthetic regions, only a limited number of 13 unique types were identified (Table 3). These numbers are minimal compared to well-known secondary metabolite producers [27,29]. They also showed limited similarity with the known BGCs, suggesting a potential for novel compounds from this genus. Among these clusters, non-ribosomal peptide synthetase (NRPS) types were the most abundant, with at least 10 such regions distributed among nine strains.

Moreover, 4 out of 10 such NRPS regions were identified in *A. aneurinilyticus* ATCC 12856^T^. One of these NRPS BGC was predicted to produce gramicidin S. Similarly, a gramicidin S BGC was also predicted in the genome of *A. migulanus*. Both these clusters exhibited limited similarity with the known gramicidin S BGC (MIBiG ID: BGC0000367) from *Brevibacillus brevis* NBRC 100599. The enzymes involved in the biosynthesis of gramicidin S from two *Aneurinibacillus* strains were identified only after manual inspection. Interestingly, no Type I and II PKS (Polyketide synthase) regions were detected in any genomes. However, the genome of each strain except *Aneurinibacillus* sp. XH2 and *A. thermoaerophilus* contained one Type III PKS region.

The biosynthetic regions found in *Aneurinibacillus* genomes also exhibited limited similarity with the known biosynthetic gene clusters (BGCs) of the MIBiG [80] database (Table S3). Only 17 regions out of 60 exhibited some similarity with the known BGCs in the MIBiG database. The percentage of similarity shared by most secondary metabolite-producing regions ranged between 8 and 50%, respectively. Region 8 with lanthipeptide encoding potential from *Aneurinibacillus* sp. XH2 showed a very high similarity of 90% with the geobacillin I (MIBiG id: BGC0000515) BGC from *Geobacillus thermodenitrificans* NG80-2^T^ [81]. Geobacillin I is an analog of lantibiotic nisin and is used as a food preservative. Geobacillin I contains seven thioether cross-links and has a broad antimicrobial spectrum against *Streptococcus dysgalactiae*. Geobacillin I comprises a gene cluster having short open reading frames for the precursor peptides. Genes present in BGC of geobacillin I include *geoAI*, *geoB*, *geoTI*, *geoC*, *geoR*, *geok*, *geol*, *geoG*, *geoE* and *geoF*.

Similarly, region 18.7 (Lassopeptide) from *A. migulanus* exhibited 80% similarity with the paeninodin (MIBiG id: BGC0001356) biosynthetic BGC from *Paenibacillus dendritiformis* C454^T^ [82]. Paeninodin is a post-translationally modified peptide (RiPP) and belongs to the class of lasso peptides, which are known for their antimicrobial and antiviral activities. They may also function as potent receptor antagonists [82,83]. Paeninodin isolated from the *Paenibacillus dendritiformis* C454 revealed a unique gene in its biosynthetic cluster, encoding a novel kinase with a peptide-tailoring function [84]. The paeninodin BGC of *Paenibacillus dendritiformis* is comprised of *padeC*, *padeA*, *padeK*, *padeB1*, *padeB2*, and *padeD* genes [84].

#### 3.4.2. CAZymes of *Aneurinibacillus*

**Figure 4:**
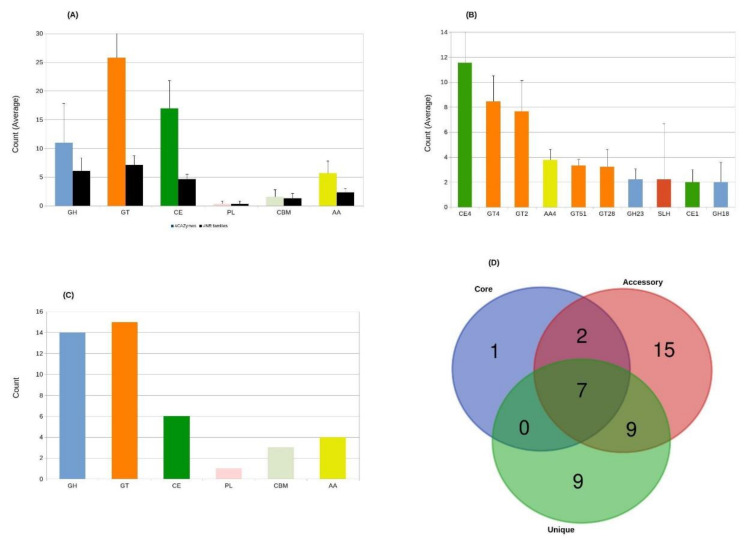
Distribution of various CAZymes in nine *Aneurinibacillus* genomes. No. of various types of CAZy genes (**A**) most abundant CAZy families (**B**) No. of unique CAZy families (**C**), and No. of CAZymes in pan-genome (**D**).

The recent reports on the possible biomass degradation capabilities of *Aneurinibacillus* [23,85] strains encouraged us to explore the potential CAZyme encoding genes within their genomes. About 551 CAZy genes, which ranged between 47 (*Aneurinibacillus* sp. XH2) and 89 (*A. tyrosinisolvens*), were found in *Aneurinibacillus* genomes. These numbers represent <2% of the proteins encoded by their genomes (Table S4). Of these 551 CAZyme encoding genes, 111 (~20% of total CAZymes) possessed a signal peptide in their amino acid sequences. Specifically, 25 lipoprotein (Sec/SPII) and 86 Sec signal peptide-containing CAZymes were found and therefore can be classified as secreted proteins (Table S5). Overall, the number of glycosyltransferases (GTs) and carbohydrate esterases (CEs) in *Aneurinibacillus* species were much higher than the glycoside hydrolases (GHs) (Figure 4A). These results are in contrast with bacteria belonging to *Streptomycetaceae* where the number of GHs is relatively higher [28]. GTs play crucial roles in oligo- and polysaccharides biosynthesis, protein glycosylation, and the formation of beneficial natural products [86]. Similarly, CEs represent diverse CAZy families that help remove O-(ester) and N-(acetyl) moieties from carbohydrates. Such enzymes have been implicated in several biological and industrial applications such as biomass degradation and drug discovery [87]. Among individual CAZy members, CE4, followed by GT4, were the two topmost abundant CAZy families identified in *Aneurinibacillus* genomes (Figure 4B). CE4 enzymes in bacteria are responsible for removing acetyl groups from chitin, chitosan, and chitooligosaccharides. These enzymes may also act on peptidoglycan and acetyl xylan [88,89,90]. A total of 104 different genes with CE4 domains were found in nine *Aneurinibacillus* genomes, of which more than 61% (64/104) possessed a signal peptide. The amino acid sequence of each of these genes exhibited notable diversity and variable sequence length. CE4 enzymes are known to have several conserved histidine (HIS) and aspartic acid (ASP) residues (Figure S2) [91]. The GT4 family, along with GT2, is one of the largest known GT families. However, GT4 members contain a GT-B fold as compared to GT-A fold of the GT2 representatives. The difference between the two folds is that in the GT-B fold, the two domains are loosely linked and face each other with the active-site lying inside the resulting cleft [86]. Overall, a limited number of unique CAZy families were observed in *Aneurinibacillus* genomes (Figure 4C).

Although, as expected, higher number of CAZymes were found in the accessory genome than the core and unique genomes. At least 7 (GT2, GT4, GT28, GH23, CE1, CE4, CE14) CAZy families were common among all the three pan-genome components. In contrast, both accessory and unique genomes consisted of multiple CAZy families specific to these groups (Table 4), whereas only one family (GT26) specific to the core genome was identified (Figure 4D). Members of the GT26 family also contain GT-B fold; however, as compared to family GT4, individuals of GT26 are inverting enzymes [86]. The accessory genome of *A. tyrosinisolvens* LL-002^T^ contained a maximum of 44 CAZy genes, out of which 7 belonged to family CE4. In contrast, only 25 CAZy genes were identified in the accessory genome of *A. soli* CB4^T^, and out of these eight were annotated as CE4 family enzymes. CE4 family enzymes were most abundant in the accessory genomes of all the strains except in the cases of *A. danicus* NBRC 102444^T^, *Aneurinibacillus* sp. XH2^T^ and *A. thermoaerophilus* L 420-91^T^. Among these three strains, the former had a higher number of GT2 enzymes, whereas the other two strains consisted of more GT4 enzymes in their accessory genomes. GT2 enzymes play roles in forming numerous β-linked polysaccharides, including cellulose, chitin, and hyaluronan [91]. The accessory genome of five (*A. soli* CB4^T^, *Aneurinibacillus* sp. UBA3580, *Aneurinibacillus* sp. XH2, *A. terranovensis* DSM 18919^T^, and *A. thermoaerophilus* L 420-91^T^) strains also contained one or two surfaces (S-) and layer homology (SLH) domains that anchor various bacterial proteins non-covalently to the cell surface [92]. In case an accessory protein contained more than one SLH domains, it was also associated with an additional domain representing carbohydrate-binding module family 54 (CBM54). Interestingly, nine SLH domains were also found in the unique genome of *A. soli* CB4^T^. However, no CBMs were linked to any of these SLH domains. Moreover, in contrast to the accessory genome, the unique genome of *A. soli* CB4^T^ has the highest number of genes with CAZy domains (including the most abundant 8 SLH domains). A single SLH domain was also identified in the unique genome of strain *A. tyrosinisolvens* LL-002^T^. SLH domains have a highly conserved TRAE motif, crucial in binding nonclassical secondary cell wall polymers (SCWPs) [93]. No singleton belonging to any CAZy family was identified in the unique gnome of *Aneurinibacillus* sp. XH2.

Additionally, the type of most abundant CAZy family in the species-specific genomes varied considerably. For example, GT4 was most common in the unique genomes of *A. danicus* NBRC 102444^T^, *A. migulanus* DSM 2895^T^, and *Aneurinibacillus* sp. UBA3580; whereas GT2 was the most abundant CAZy domain in the unique genomes of *A. thermoaerophilus* L 420-91^T^ and *A. tyrosinisolvens* LL-002^T^. In contrast, CE4, one of the most frequently occurring domains in the accessory genomes of several strains, was only abundant in the species-specific genome of *A. terranovensis* DSM 18919^T^.

#### 3.4.3. Genes Involved in Heavy Metal and Antibiotic Resistance

**Figure 5:**
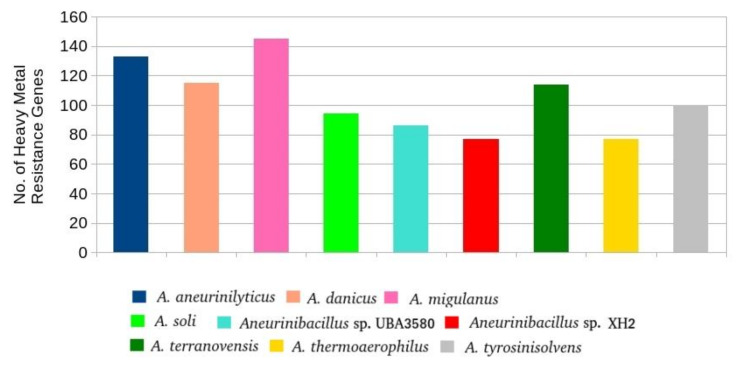
Distribution of heavy metal resistance genes in *Aneurinibacillus* genomes.

Bacteria utilize metal accumulation or biotransformation for metal detoxification [94,95]. Most heavy metals (especially at higher concentrations) can be fatal for all living organisms and may also impact the whole ecosystem [96,97]. Many genera of the *Paenibacillaceae* family have been reported to show resistance towards heavy metals. For example, *Paenibacillus* *polymyxa* can be effectively used as an adsorbent to remove copper and nickel from aqueous solutions [98]. Similarly, *Paneibacillus* sp. RM, isolated from the roots of *Tridax procumbens*, has shown high resistance to copper, zinc, and arsenic [99]. Among *Aneurinibacillus*, strains of *A. aneurinilyticus* isolated from groundwater can oxidize arsenite to less toxic arsenate, and therefore have potential for the bioremediation of arsenic [24]. Arsenic exists in organic and inorganic forms and is widely distributed in the environment. Inorganic arsenic exists mainly in trivalent (AsIII) and pentavalent (AsV) forms and is considered highly toxic to human health. Among them, trivalent compounds are more harmful than pentavalent forms [100,101,102]. The prokaryotic genomes are dominated by multiple *ars* operons with several genes and various combinations, including their accessory plasmids and transposons. Additional genes have recently been discovered in addition to these accepted common *ars* gene clusters [103,104]. Our results show that the arsenic reductase enzyme (*arsC*) was identified in almost all *Aneurinibacillus* strains except *A. aneurinilyticus* and *Aneurinibacillus* sp. UBA3580. It has been reported that *arsC* can reduce (AsIII) into (AsV), thus converting a more toxic form of arsenic into a less toxic form [105,106]. Moreover, in *E. coli*, it has been shown that resistance towards arsenic is regulated through the activity of *arsC* [107]. There are several common pathways for arsenic resistance in prokaryotes. Under aerobic conditions, with the assistance of *pstA*, *pstB*, *pstC* and *PhoS*, As(V) enters the cell through a phosphate uptake mechanism and is then reduced by *arsC* to As(III) [103]. Some of these genes (*pstA*, *pstB*, *pstC*) were identified in all *Aneurinibacillus* strains. Thus we can conclude from our results that *Aneurinibacillus* strains have several common pathways towards arsenic resistance akin to other bacterial species. The *arsM* gene, accountable for organoarsenical detoxification, encodes an As(III) S-adenosylmethionine methyltransferase and was identified in *A. aneurinilyticus*, *A. danicus*, *A. soli*, *Aneurinibacillus sp. UBA3580* and *A. terranovensis*. *arsM* genes in some microbes encode ArsM As(III) S-adenosylmethionine methyltransferases that transform As(III) into the considerably more toxic organoarsenical methylarsenite (MAs(III)) and may be responsible for animal carcinogenesis [108]. Various *Aneurinibacillus* strains have also shown resistance against multiple heavy metals [25]. In this work, several genes related to heavy metal resistance were identified in each of the *Aneurinibacillus* strains and ranged between 77 (*Aneurinibacillus* sp. XH2 and *A. thermoaerophilus*) and 145 (*A. migulanus*) (Figure 5). These numbers are promising and even higher in some cases as compared to some known heavy metal resistant bacteria [29]. Interestingly, the top two strains (*A. aneurinilyticus* and *A. migulanus*) with a maximum number of heavy metal resistance genes also have the most number of BGCs, again suggesting a strong correlation between these two characteristics of bacteria [29]. Among these genes, *zraR*/*hydH* (transcriptional regulatory protein), *corR* (sigma-54 dependent DNA-binding response regulator), and *copR* (transcriptional activator protein) were the most abundant heavy metal resistance genes found in all nine *Aneurinibacillus* genomes (Table 5 and Table S6). *zraR*/*hydH* is involved in zinc (Zn) tolerance [109] whereas the latter two have been implicated in copper (Cu) resistance [110,111]. In addition to these 3 genes, 23 additional genes were found in each of the nine genomes, making them the core set of heavy metal resistance or antibacterial biocide genes in *Aneurinibacillus*. These include genes for arsenic (As), chromium (Cr), iron (Fe), molybdenum (Mo), nickel (Ni), tellurium (Te), tungsten (W), and antibacterial biocides such as triclosan. However, their number varied in the genomes of each strain. Some of these genes, such as *recG* (ATP-dependent DNA helicase), shows resistance against multiple metals [112]. In addition to the core set of heavy metal resistance genes, the genomes of some strains contained additional genes with roles in metal resistance. For example, four *terD* genes were identified only in the genome of *A. terranovensis*. This gene encodes a tellurium resistance protein essential for tellurium resistance [113]. A single *arrB*, *merA*, and *nixA* gene, which are implicated in resistance against As, mercury (Hg), and Ni, respectively, were also identified only in the genome of *A. terranovensis*. However, other genes that exhibit resistance against these metals were found in other genomes. For example, arsenic resistant *pstA*/*C*/*S* genes [114,115] were identified in all the nine genomes and represented the core set of heavy metal resistance genes in *Aneurinibacillus*. Similarly, two genes (*fbpA* and *fbpB*) involved in the resistance against Fe and gallium (Ga) [116] were specifically detected only in the genome of *A. aneurinilyticus*.

An increasing number of bacterial species develop clinical resistance to antimicrobial agents, and the underlying mechanisms of their resistance are continuously investigated [116]. A family of transmembrane proteins, often referred to as drug resistance translocases, are involved in removing such antibiotics from the cells [117]. Such systems were also found in *Aneurinibacillus* genomes. Enoyl-[acyl-carrier-protein] reductase (NADH) enzyme encoded by the *fabL* gene was identified in all the nine genomes. The genomes of *A. danicus*, *A. migulanus*, and *A. terranovensis* contained two copies of this gene, whereas only one copy of *fabL* was detected in the remaining genomes. *fabL* catalyzes the reduction of a carbon–carbon double bond in an enoyl group covalently linked to an acyl carrier protein (ACP). This enzyme is known to show resistance against triclosan, a compound with a broad range of antibacterial and antifungal activities [118]. In contrast, two *vcaM* genes that encode an ABC multidrug efflux pump were identified only in the *A. aneurinilyticus* genome. Based on the amino acid sequence, vcaM exhibits similarity to human multidrug resistance proteins P-glycoprotein (MDR1) and lactococcal LmrA. This gene has shown resistance towards several structurally unrelated drugs, such as tetracycline, norfloxacin, and ciprofloxacin, including others [119]. Similarly, a single bicyclomycin resistance protein encoded by the *BcR* gene [120] was found only in the *A. terranovensis* genome. These data suggest that while there are a highly conserved set of metal and antibacterial-resistant genes in the genus *Aneurinibacillus*, some genes are uniquely present in the genomes of specific strains.

## 4. Conclusions

This work is the first comprehensive genome-based analyses of *Aneurinibacillus*, which highlighted the significance of this genus with industrial implications, especially with a focus on their bioremediation, biodegradation, and biosynthetic capabilities. In conclusion, we observe the presence of regions that may represent biosynthetic gene clusters in all the *Aneurinibacillus* genomes analyzed in this work. However, these regions exhibited minimal similarity with the known biosynthetic gene clusters, suggesting that the potential SMs produced by the genus *Aneurinibacillus* might be novel with a broad range of possible bioactivities. Several CAZymes, especially glycosyltransferases and carbohydrate esterases, which have roles in the biosynthesis and degradation of a variety of structures, were also identified. Moreover, the diverse types of genes and enzymes involved in heavy metal resistance highlighted in this study would offer initial clues to further explore the genus for its full potential in industrial applications.

## Figures and Tables

**Table 1:** Overview of the general genomic features of nine *Aneurinibacillus* strains analyzed in this work.

Genome Name	BioProject Accession	No. of Contigs	Genome Size (Mb)	GC Content	Patric CDS	Pseudogenes	tRNA (rRNA)	No. of Accessory Genes	No. of Unique Genes	Genome Completeness (%)	Isolation Source
*Aneurinibacillus aneurinilyticus* ATCC 12856^T^	PRJNA38313	395	5.30	43.2	5594	184	39/4	2781	1545	99.1	Gastrointestinal tract
*Aneurinibacillus danicus* NBRC 102444^T^	PRJDB6144	297	4.42	46.67	4912	123	30/3	2505	743	99.3	NA
*Aneurinibacillus migulanus* DSM 2895^T^	PRJNA291108	28	6.35	42.94	7259	504	85/12	2850	969	99.5	Soil
*Aneurinibacillus soli* CB4^T^	PRJDB4401	1	4.12	45.89	4221	54	169/51	1479	982	98.0	NA
*Aneurinibacillus* sp. UBA3580	PRJNA348753	137	3.56	47.15	3971	NA	40/NA	2066	326	91.5	Topsoil
*Aneurinibacillus* sp. XH2	PRJNA287204	1	3.67	44.86	4051	85	114/34	1865	88	98.7	Oil produced water
*Aneurinibacillus terranovensis* DSM 18919^T^	PRJNA188818	118	4.24	44.9	4500	186	75/9	1353	1049	98.7	Geothermal soil
*Aneurinibacillus thermoaerophilus* L 420-91^T^	PRJEB15926	103	3.66	44.51	4176	152	61/13	1903	182	99.1	NA
*Aneurinibacillus tyrosinisolvens* LL-002^T^	PRJDB3821	136	5.69	44.54	6607	368	123/3	1806	1725	95.1	Organics- and methane-rich seafloor sediment

NA = Not Applicable.

**Table 2:** Pairwise comparisons of dDDH values between nine *Aneurinibacillus* genomes. The values are based on three different GBDP formulas: d0 (length of all HSPs divided by total genome length); d4 (sum of all identities found in HSPs divided by overall HSP length); d6 (sum of all identities found in HSPs divided by total genome length). Genome of *Brevibacillus brevis* NCTC 2611^T^ was used as an out-group.

Query Strain	Subject Strain	dDDH (d0, in %)	dDDH (d4, in %)	dDDH (d6, in %)
*Aneurinibacillus* sp. XH2	*Aneurinibacillus thermoaerophilus* L 420-91^T^	94	98.4	96.5
*Aneurinibacillus danicus* NBRC 102444^T^	*Aneurinibacillus* sp. UBA3580	68.5	95.5	75.3
*Aneurinibacillus aneurinilyticus* ATCC 12856^T^	*Aneurinibacillus migulanus* DSM 2895^T^	44.9	35.8	42.3
*Aneurinibacillus* sp. XH2	*Brevibacillus brevis* NCTC 2611^T^	12.8	30.9	13.2
*Aneurinibacillus danicus* NBRC 102444^T^	*Brevibacillus brevis* NCTC 2611^T^	12.7	28.3	13.1
*Aneurinibacillus soli* CB4^T^	*Brevibacillus brevis* NCTC 2611^T^	12.9	27.5	13.3
*Aneurinibacillus migulanus* DSM 2895^T^	*Brevibacillus brevis* NCTC 2611^T^	12.7	26.4	13.1
*Aneurinibacillus tyrosinisolvens* LL-002^T^	*Brevibacillus brevis* NCTC 2611^T^	12.6	24.9	13
*Aneurinibacillus danicus* NBRC 102444^T^	*Aneurinibacillus terranovensis* DSM 18919^T^	13.4	24	13.7
*Aneurinibacillus aneurinilyticus* ATCC 12856^T^	*Brevibacillus brevis* NCTC 2611^T^	12.7	23.3	13.1
*Aneurinibacillus* sp. UBA3580	*Aneurinibacillus terranovensis* DSM 18919^T^	13.2	23.1	13.6
*Aneurinibacillus thermoaerophilus* L 420-91^T^	*Brevibacillus brevis* NCTC 2611^T^	12.5	23.1	12.9
*Aneurinibacillus terranovensis* DSM 18919^T^	*Brevibacillus brevis* NCTC 2611^T^	12.5	23	12.9
*Aneurinibacillus soli* CB4^T^	*Aneurinibacillus tyrosinisolvens* LL-002^T^	13.5	21.8	13.8
*Aneurinibacillus danicus* NBRC 102444^T^	*Aneurinibacillus migulanus* DSM 2895^T^	18	21.7	17.7
*Aneurinibacillus danicus* NBRC 102444^T^	*Aneurinibacillus* sp. XH2	17.2	21.6	17.1
*Aneurinibacillus migulanus* DSM 2895^T^	*Aneurinibacillus soli* CB4^T^	13.8	21.5	14.1
*Aneurinibacillus migulanus* DSM 2895^T^	*Aneurinibacillus* sp. UBA3580	17.6	21.4	17.4
*Aneurinibacillus aneurinilyticus* ATCC 12856^T^	*Aneurinibacillus danicus* NBRC 102444^T^	17.8	21.4	17.5
*Aneurinibacillus aneurinilyticus* ATCC 12856^T^	*Aneurinibacillus* sp. UBA3580	17.3	21.4	17.1
*Aneurinibacillus* sp. UBA3580	*Aneurinibacillus* sp. XH2	16.5	21.3	16.4
*Aneurinibacillus danicus* NBRC 102444^T^	*Aneurinibacillus thermoaerophilus* L 420-91^T^	16.8	21.2	16.7
*Aneurinibacillus soli* CB4^T^	*Aneurinibacillus* sp. XH2	14.3	21	14.5
*Aneurinibacillus* sp. UBA3580	*Aneurinibacillus thermoaerophilus* L 420-91^T^	16.1	20.7	16.1
*Aneurinibacillus* sp. XH2	*Aneurinibacillus tyrosinisolvens* LL-002^T^	13.4	20.6	13.7
*Aneurinibacillus terranovensis* DSM 18919^T^	*Aneurinibacillus tyrosinisolvens* LL-002^T^	13.5	20.4	13.8
*Aneurinibacillus migulanus* DSM 2895^T^	*Aneurinibacillus* sp. XH2	15.6	20.3	15.6
*Aneurinibacillus aneurinilyticus* ATCC 12856^T^	*Aneurinibacillus soli* CB4^T^	14.1	20.2	14.3
*Aneurinibacillus* sp. XH2	*Aneurinibacillus terranovensis* DSM 18919^T^	13.3	20.1	13.6
*Aneurinibacillus soli* CB4^T^	*Aneurinibacillus terranovensis* DSM 18919^T^	13.1	20.1	13.4
*Aneurinibacillus aneurinilyticus* ATCC 12856^T^	*Aneurinibacillus* sp. XH2	15.6	19.9	15.6
*Aneurinibacillus migulanus* DSM 2895^T^	*Aneurinibacillus tyrosinisolvens* LL-002^T^	13.3	19.8	13.6
*Aneurinibacillus migulanus* DSM 2895^T^	*Aneurinibacillus thermoaerophilus* L 420-91^T^	15.2	19.7	15.3
*Aneurinibacillus terranovensis* DSM 18919^T^	*Aneurinibacillus thermoaerophilus* L 420-91^T^	13.3	19.5	13.6
*Aneurinibacillus danicus* NBRC 102444^T^	*Aneurinibacillus tyrosinisolvens* LL-002^T^	13.6	19.5	13.9
*Aneurinibacillus danicus* NBRC 102444^T^	*Aneurinibacillus soli* CB4^T^	14.3	19.4	14.5
*Aneurinibacillus aneurinilyticus* ATCC 12856^T^	*Aneurinibacillus thermoaerophilus* L 420-91^T^	15.4	19.3	15.4
*Aneurinibacillus migulanus* DSM 2895^T^	*Aneurinibacillus terranovensis* DSM 18919^T^	13	19.1	13.4
*Aneurinibacillus* sp. UBA3580	*Aneurinibacillus tyrosinisolvens* LL-002^T^	13.5	18.8	13.8
*Aneurinibacillus thermoaerophilus* L 420-91^T^	*Aneurinibacillus tyrosinisolvens* LL-002^T^	13.2	18.7	13.5
*Aneurinibacillus aneurinilyticus* ATCC 12856^T^	*Aneurinibacillus tyrosinisolvens* LL-002^T^	13.5	18.5	13.8
*Aneurinibacillus aneurinilyticus* ATCC 12856^T^	*Aneurinibacillus terranovensis* DSM 18919^T^	13	18.4	13.4
*Aneurinibacillus* sp. UBA3580	*Brevibacillus brevis* NCTC 2611^T^	12.5	18	12.9
*Aneurinibacillus soli* CB4^T^	*Aneurinibacillus* sp. UBA3580	13.9	18	14.1
*Aneurinibacillus soli* CB4^T^	*Aneurinibacillus thermoaerophilus* L 420-91^T^	13.9	17.9	14.1

**Table 3:** Distribution of different types of biosynthetic gene clusters in the genomes of various *Aneurinibacillus* strains.

S. No.	Cluster Type	*A. aneurinilyticus* ATCC 12856^T^	*A. danicus* NBRC 102444^T^	*A. migulanus* DSM 2895^T^	*A. soli* CB4^T^	*Aneurinibacillus* sp. UBA3580	*Aneurinibacillus* sp. XH2	*A. terranovensis* DSM 18919^T^	*A. thermoaerophilus* L 420-91^T^	*A. tyrosinisolvens* LL-002^T^	Total
1.	Arylpolyene	0	0	0	0	0	1	0	1	0	2
2.	Bacteriocin	2	1	1	0	1	1	0	1	2	9
3.	Betalactone	1	1	1	0	0	1	0	1	1	6
4.	Hserlactone	0	0	0	0	0	1	0	1	0	2
5.	Lanthipeptide	0	0	0	0	0	1	0	1	1	3
6.	LAP/bacteriocin	1	1	0	0	0	1	0	1	0	4
7.	Lassopeptide	0	0	1	0	0	0	0	0	0	1
8.	NRPS	4	0	2	2	0	0	1	0	1	10
9.	Phosphonate	0	0	1	0	0	0	0	0	0	1
10.	Sactipeptide	0	0	0	0	0	1	0	1	0	2
11.	Siderophore	0	1	1	0	1	1	0	1	0	5
12.	T3PKS	1	1	1	1	1	0	1	0	1	7
13.	Terpene	1	1	2	2	1	0	0	0	1	8
	Total	10	6	10	5	4	8	2	8	7	60

**Table 4:** Number of specific CAZy families for accessory, core and unique genomes of *Aneurinibacillus*. In case of multiple activities, only the first three are reported.

Pan-Genome	CAZy Family	Main Activity	*A. aneurinilyticus* ATCC 12856^T^	*A. danicus* NBRC 102444^T^	*A. migulanus* DSM 2895^T^	*A. soli* CB4^T^	*Aneurinibacillus* sp. UBA3580	*Aneurinibacillus* sp. XH2	*A. terranovensis* DSM 18919^T^	*A. thermoaerophilus* L 420-91^T^	*A. tyrosinisolvens* LL-002^T^
**Accessory**	**CBM48**	Glycogen-binding function, Beta subunit (glycogen-binding) of AMP-activated protein kinases	-	1	-	-	1	-	1	-	1
	**CBM54**	Binding to xylan yeast cell wall	1	-	-	1	-	-	1	-	-
	**CE7**	Acetyl xylan esterase Cephalosporin-C deacetylase	1	-	1	-	-	-	-	-	-
	**GH2**	Β-Galactosidase, β-mannosidase, β-glucuronidase	1	1	1	1	1	-	-	-	1
	**GH13**	α-amylase pullulanase, Cyclomaltodextrin glucanotransferase	-	2	-	-	2	-	3	-	2
	**GH25**	Lysozyme	2	-	-	-	-	-	-	-	2
	**GH31**	α-glucosidase, α-galactosidase, α-mannosidase	1	-	-	-	1	-	-	-	1
	**GH73**	Lysozyme, mannosyl-glycoprotein endo-β-N acetylglucosaminidase peptidoglycan hydrolase with endo-β-N-acetylglucosaminidase specificity	2	**1**	-	-	1	1	-	1	1
	**GH88**	d-4,5-unsaturated β-glucuronyl hydrolase	-	1	-	-	1	-	-	-	-
	**GT1**	UDP-glucuronosyltransferase zeatin O-β-xylosyltransferase, Hydroxyacylsphingosine 1-β-galactosyltransferase	1	-	-	-	-	-	-	-	1
	**GT5**	UDP-Glc, glycogen glucosyltransferase ADP-Glc, Starch glucosyltransferase NDP-Glc, Starch glucosyltransferase	-	1	-	-	1	-	1	-	1
	**GT35**	Glycogen, Starch phosphorylase	-	1	-	-	1	-	1	-	1
	**GT89**	β-D-arabinofuranosyl-1-monophosphoryldecaprenol arabinan β-1,2-arabinofuranosyltransferase	1	-	1	-	-	-	-	-	-
	**GT94**	GDP-Man: GlcA-β-1,2-Man-α-1,3-Glc-β-1, 4-Glc-α-1-PP-undecaprenol β-1, 4-mannosyltransferase	-	-	-	-	-	1	-	1	-
	**PL17**	Alginate lyase, Oligoalginate lyase	-	1	-	-	1	-	1	-	-
**Core**	**GT26**	UDP-ManNAcA: β-N-acetyl mannosaminuronyltransferase, UDP-ManNAc: β-N-acetyl-mannosaminyltransferase UDP-Glc: β-1,4-glucosyltransferase	1								
**Unique**	**AA7**	Glucooligosaccharide oxidase, Chitooligosaccharide oxidase	-	-	-	-	1	-	-	-	-
	**GH8**	d-4,5-unsaturated β-glucuronyl hydrolase	-	-	-	-	-	-	-	-	1
	**GH32**	Invertase, endo-inulinase, β-2,6-fructan 6 levanbiohydrolase	-	-	-	-	-	-	1	-	-
	**GH94**	Cellobiose phosphorylase, laminaribiose phosphorylase, Cellodextrin phosphorylase	-	-	-	-	-	-	1	-	-
	**GH126**	α-amylase	-	-	-	1	-	-	-	-	-
	**GT27**	Polypeptide α-N-acetylgalactosaminyl transferase	-	1	-	-	-	-	-	-	-
	**GT32**	α-1,6-mannosyltransferase, α-1,4-N-acetylglucosaminyltransferase, α-1,4-N-acetylgalactosaminyltransferase	-	1	-	-	-	-	-	-	-
	**GT83**	Undecaprenyl phosphate-α-L-Ara4N, 4-amino-4-deoxy-β-L-arabinosyltransferase, Dodecaprenyl phosphate-β-galacturonic acid, lipopolysaccharide core α-galacturonosyl transferase	-	-	-	-	-	-	1	-	-
	**GT84**	Cyclic β-1,2-glucan synthase	-	-	-	-	-	-	1	-	-

**Table 5:** Top 20 most abundant heavy metal resistance genes found in the genomes of nine *Aneurinibacillus* strains.

Gene	Description	Type	Resistance	*A. aneurinilyticus* ATCC 12856^T^	*A. danicus* NBRC 102444^T^	*A. migulanus* DSM 2895^T^	*A. soli* CB4^T^	*Aneurinibacillus* sp. UBA3580	*Aneurinibacillus* sp. XH2	*A. terranovensis* DSM 18919^T^	*A. thermoaerophilus* L 420-91^T^	*A. tyrosinisolvens* LL-002^T^
*zraR/hydH*	Transcriptional regulatory protein ZraR	Regulator	Zinc (Zn)	21	16	20	6	9	8	14	8	14
*corR*	Sigma-54 dependent DNA-binding response regulator	Regulator	Copper (Cu)	8	12	16	2	9	8	12	6	10
*copR*	Transcriptional activator protein	Regulator	Copper (Cu)	5	5	5	5	4	2	7	2	4
*terD*	Tellurium resistance protein	Unknown	Tellurium (Te)	0	0	0	0	0	0	4	0	0
*mdeA*	Methionine gamma-lyase	Enzyme	Cetrimide (CTM), Benzylkonium Chloride (BAC), Hoechst 33342, Ethidium Bromide, Rhodamine 6G, Acriflavine, Tetraphenylphosphonium (TPP), Chlorhexidine, Crystal Violet, Dequalinium, Pentamidine, Pyronin Y	4	5	5	4	5	2	2	2	3
*chtR*	DNA-binding response regulator	Regulator	Chlorhexidine	5	0	8	1	0	1	0	1	0
*fecD*	Fe(3+) dicitrate transport system permease protein	Enzyme	Nickel (Ni), Cobalt (Co)	2	3	5	0	2	3	0	3	0
*merE*	Mercuric resistance protein	Membrane Transporter	Mercury (Hg)	3	3	2	5	1	0	5	0	2
*mntR*	Manganese transport regulator	Regulator	Manganese (Mn), Magnesium (Mg)	0	0	0	2	0	0	0	0	4
*ruvB*	ATP-dependent DNA helicase	Enzyme	Chromium (Cr), Tellurium (Te), Selenium (Se)	3	3	3	3	3	3	3	3	3
*fecE*	Fe(3+) dicitrate transport ATP-binding protein	Membrane Transporter	Nickel (Ni), Cobalt (Co)	5	4	4	1	2	3	1	3	1
*nikD*	Nickel import ATP-binding protein	Binding protein	Nickel (Ni)	2	0	4	2	0	0	2	0	0
*baeR*	Member of the two-component regulatory system BaeS/BaeR	Regulator	Zinc (Zn), Tungsten (W), Sodium Deoxycholate (SDC)	2	1	3	4	1	2	4	2	2
*cpxR*	Response regulator of stress-related two-component regulatory system.	Regulator	Hydrogen Peroxide (H2O2), Benzylkonium Chloride (BAC), Chlorhexidine	2	2	4	3	2	2	2	2	2
*soda*	Mn-dependent superoxide dismutase	Enzyme	Selenium (Se), Hydrogen Peroxide (H2O2)	3	2	2	3	1	2	2	3	1
*Can*	Aconitate hydratase	Enzyme	Iron (Fe)	2	2	2	2	2	2	2	2	2
*arsT*			Arsenic (As)	2	2	2	2	2	2	2	2	2
*irlR*	Transcriptional activator protein	Regulator	Cadmium (Cd), Zinc (Zn)	2	0	0	0	0	0	3	0	1
*lmrS*	LmrS is an efflux protein capable of extruding multiple and structurally unrelated antimicrobial compounds.	Efflux	Tetraphenylphosphonium (TPP), Sodium Dodecyl Sulfate (SDS), Ethidium Bromide, Cetrimide (CTM)	2	0	2	0	0	0	1	0	3
*vcaM*	ABC transporter	Binding protein	Ethidium Bromide, Rhodamine 6G, 4,6-diamidino-2-phenylindole (DAPI), Tetraphenylphosphonium (TPP), Acridine Orange	2	0	0	0	0	0	0	0	0

## Data Availability

The data presented in this study are available within the article. If required, any additional data is available on request from the authors.

## Supplementary Materials

The following are available online at https://www.mdpi.com/2076-2607/9/3/499/s1, Figure S1: Multiple sequence alignment of three selenophosphate synthase (SPS) enzymes identified only in the genomes of *A. soli*, *A. terranovensis*, and *A. tyrosinisolvens*, respectively. Known SPS sequences from *Homo sapiens*, *Aquifex aeolicus* and *E. coli* are used as reference sequences. Four highly conserved magnesium binding aspartic acid (D) residues and a conserved asparagine (N) at the active site are shown within the boxes, Figure S2: Multiple sequence alignment of representative CE4 domain containing CAZymes found in the *Aneurinibacillus* genomes. Reference sequences of known CE4 enzymes are indicated with red circles where as highly conserved metal binding aspartic acid (D), and two histidine (H) residues are shown within the boxes, Table S1: The results of genome completeness of nine *Aneurinibacillus* strains based on the BUSCO search against 450 bacillales core genes (bacillales_odb10 lineage). Table S2: List of enzymes specific to each pan-genome component of the *Aneurinibacillus* strains. Table S3: Distribution of regions with biosynthetic potential in *Aneurinibacillus*, Table S4: Distribution of CAZymes and their various families in *Aneurinibacillus*. Values in parenthesis (column 3–8) represent different types of CAZy families, Table S5: List of all CAZy genes identified in this work. Some genes have more than one CAZy domain, Table S6: Complete list of heavy metal resistance genes found in 9 *Aneurinibacillus* strains.
