# Supplementary material for: Genome-Driven Discovery of Enzymes with Industrial Implications from the Genus Aneurinibacillus
Source: Microorganisms. 2021 Feb 26;9(3):499. doi: 10.3390/microorganisms9030499 (PMC7996765; doi:10.3390/microorganisms9030499)
Supplement: Supplementary file 1 [file microorganisms-09-00499-s001.zip › microorganisms-1057738-supplementary.docx]

**Supplementary Material**

Genome-Driven Discovery of Enzymes with Industrial Implications from the Genus *Aneurinibacillus*

Majid Rasool Kamli ^1,2,*^, Nada A.Y. Alzahrani ^2^, Nahid H. Hajrah ^1^, Jamal S.M. Sabir ^1,2^ and Adeel Malik ^3,*^

^1^ Department of Biological Sciences, Faculty of Science, King Abdulaziz University (KAU), Jeddah, 21589, Saudi Arabia; mkamli@kau.edu.sa (M.R.K.); nhajrah260@gmail.com (N.H.H.); jsabir2622@gmail.com (J.S.M.S.)

^2^ Center of excellence in Bionanoscience Research, King Abdulaziz University (KAU), Jeddah, Saudi Arabia; nayalzahrani@kau.edu.sa (N.A.Y.A.)

^3^ Institute of Intelligence Informatics Technology, Sangmyung University, Seoul 30316, Republic of Korea; adeel@procarb.org (A.M.)

* Correspondence: adeel@procarb.org (A.M.); mkamli@kau.edu.sa (M.R.K.)


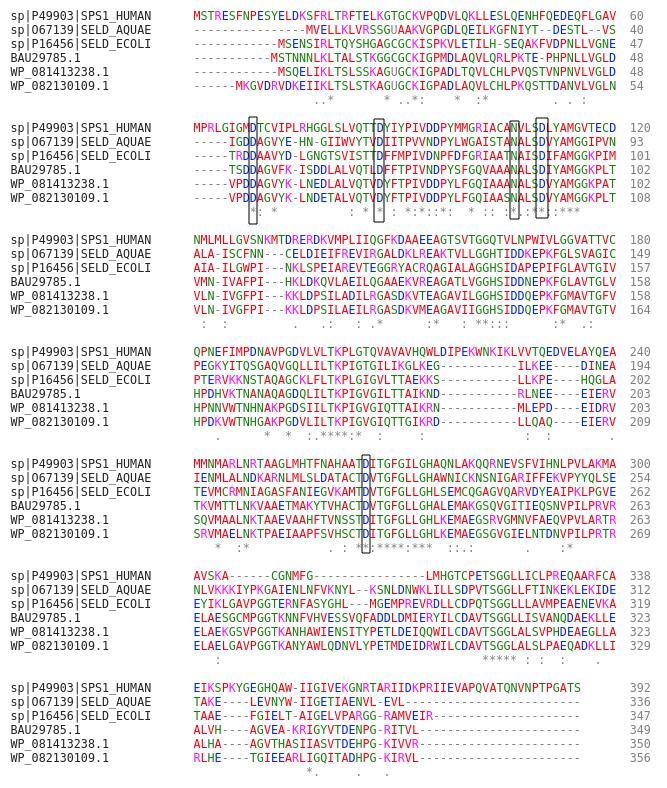


**Figure S1: Multiple sequence alignment of three selenophosphate synthase (SPS) enzymes identified only in the genomes of *A. soli*, *A. terranovensis*, and *A. tyrosinisolvens,* respectively.** Known SPS sequences from *Homo sapiens*, Aquifex aeolicus and E. coli are used as reference sequences. Four highly conserved magnesium binding aspartic acid (D) residues and a conserved asparagine (N) at the active site are shown within the boxes.


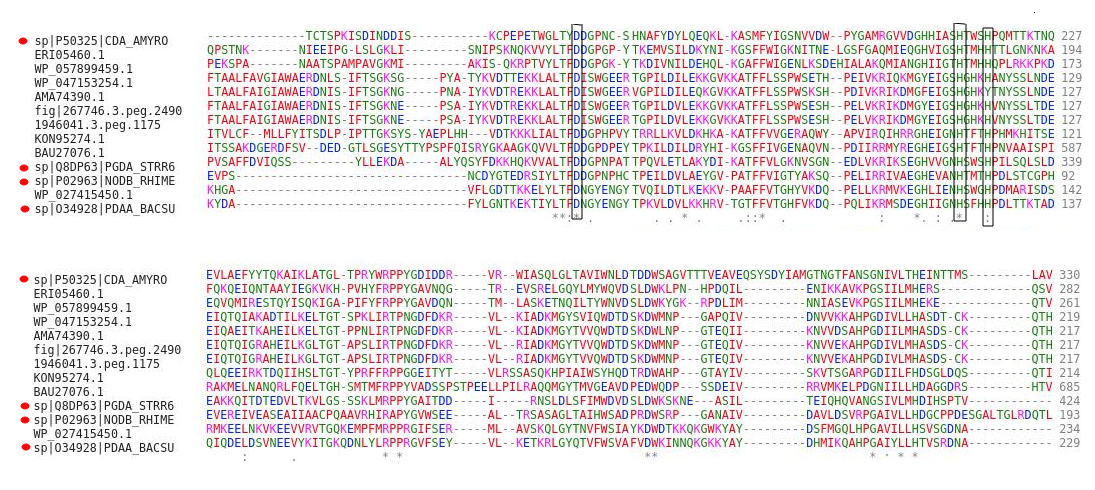


**Figure S2: Multiple sequence alignment of representative CE4 domain containing CAZymes found in the *Aneurinibacillus* genomes.** Reference sequences of known CE4 enzymes are indicated with red circles where as highly conserved metal binding aspartic acid (D), and two histidine (H) residues are shown within the boxes.

**Table S1.** The results of genome completeness of nine *Aneurinibacillus* strains based on the BUSCO search against 450 bacillales core genes (bacillales_odb10 lineage).

|  | **Percent (%)** | | | | |
| --- | --- | --- | --- | --- | --- |
| **Genome Name** | **Overall**  **Completeness** | **Complete**  **and single-copy (S)** | **Complete**  **and duplicated**  **BUSCOs (D)** | **Fragmented BUSCOs (F)** | **Missing BUSCOs (M)** |
| *Aneurinibacillus aneurinilyticus* ATCC 12856^T^ | 99.1 | 99.1 | 0.0 | 0.4 | 0.5 |
| *Aneurinibacillus danicus* NBRC 102444^T^ | 99.3 | 98.9 | 0.4 | 0.4 | 0.3 |
| *Aneurinibacillus migulanus* DSM 2895^T^ | 99.5 | 98.2 | 1.3 | 0.0 | 0.5 |
| *Aneurinibacillus soli* CB4^T^ | 98.0 | 97.8 | 0.2 | 0.2 | 1.8 |
| *Aneurinibacillus* sp. UBA3580 | 91.5 | 91.3 | 0.2 | 2.9 | 5.6 |
| *Aneurinibacillus* sp. XH2 | 98.7 | 98.7 | 0.0 | 0.2 | 1.1 |
| *Aneurinibacillus terranovensis* DSM 18919^T^ | 98.7 | 98.7 | 0.0 | 0.2 | 1.1 |
| *Aneurinibacillus thermoaerophilus* L 420-91^T^ | 99.1 | 99.1 | 0.0 | 0.0 | 0.9 |
| *Aneurinibacillus tyrosinisolvens* LL-002^T^ | 95.1 | 92.4 | 2.7 | 2.9 | 2.0 |

**Table S2: List of enzymes specific to each pan-genome component of the *Aneurinibacillus* strains.**

| **Pan-genome** | **EC** | **Enzyme name** | Reaction catalysed |
| --- | --- | --- | --- |
| Core | EC:1.3.5.1 | Succinate dehydrogenase | Succinate + a quinone <=> fumarate + a quinol |
|  | EC:1.3.5.4 | Fumarate reductase (quinol) | Succinate + a quinone <=> fumarate + a quinol |
|  | EC:1.4.4.2 | Glycine dehydrogenase (aminomethyl-transferring) | Glycine + [glycine-cleavage complex H protein]-N(6)-lipoyl-L-lysine <=> [glycine-cleavage complex H protein]-S-aminomethyl-N(6)-dihydrolipoyl-L-lysine + CO(2) |
|  | EC:1.5.5.2 | Proline dehydrogenase | L-proline + a quinone <=> (S)-1-pyrroline-5-carboxylate + a quinol |
|  | EC:1.17.1.8 | 4-hydroxy-tetrahydrodipicolinate reductase | (S)-2,3,4,5-tetrahydropyridine-2,6-dicarboxylate + NAD(P)(+) + H(2)O <=> (2S,4S)-4-hydroxy-2,3,4,5-tetrahydrodipicolinate + NAD(P)H |
|  | EC:1.17.7.3 | (E)-4-hydroxy-3-methylbut-2-enyl-diphosphate synthase (flavodoxin) | (E)-4-hydroxy-3-methylbut-2-en-1-yl diphosphate + H(2)O + oxidized flavodoxin <=> 2-C-methyl-D-erythritol 2,4-cyclodiphosphate + reduced flavodoxin |
|  | EC:1.17.7.4 | 4-hydroxy-3-methylbut-2-enyl diphosphate reductase | Isopentenyl diphosphate + 2 oxidized ferredoxin [iron-sulfur] cluster + H(2)O <=> (E)-4-hydroxy-3-methylbut-2-en-1-yl diphosphate + 2 reduced ferredoxin [iron-sulfur] cluster + 2 H(+)  Dimethylallyl diphosphate + 2 oxidized ferredoxin [iron-sulfur] cluster + H(2)O <=> (E)-4-hydroxy-3-methylbut-2-en-1-yl diphosphate + 2 reduced ferredoxin [iron-sulfur] cluster + 2 H(+) |
|  | EC:1.17.99.6 | Epoxyqueuosine reductase | Queuosine(34) in tRNA + acceptor + H(2)O <=> epoxyqueuosine(34) in tRNA + reduced acceptor |
|  | EC:2.1.2.1 | Glycine hydroxymethyltransferase | 5,10-methylenetetrahydrofolate + glycine + H(2)O <=> tetrahydrofolate + L-serine |
|  | EC:2.1.2.3 | Phosphoribosylaminoimidazolecarboxamide formyltransferase | 10-formyltetrahydrofolate + 5-amino-1-(5-phospho-D-ribosyl)imidazole-4-carboxamide <=> tetrahydrofolate + 5-formamido-1-(5-phospho-D-ribosyl)imidazole-4-carboxamide |
|  | EC:2.1.2.9 | Methionyl-tRNA formyltransferase | 10-formyltetrahydrofolate + L-methionyl-tRNA(fMet) <=> tetrahydrofolate + N-formylmethionyl-tRNA(fMet) |
|  | EC:2.1.2.10 | Aminomethyltransferase | Protein]-S(8)-aminomethyldihydrolipoyllysine + tetrahydrofolate <=> [protein]-dihydrolipoyllysine + 5,10-methylenetetrahydrofolate + NH(3) |
|  | EC:2.1.2.11 | 3-methyl-2-oxobutanoate hydroxymethyltransferase | 5,10-methylenetetrahydrofolate + 3-methyl-2-oxobutanoate + H(2)O <=> tetrahydrofolate + 2-dehydropantoate |
|  | EC:2.4.99.17 | S-adenosylmethionine:tRNA ribosyltransferase-isomerase | S-adenosyl-L-methionine + 7-aminomethyl-7-carbaguanosine(34) in tRNA <=> L-methionine + adenine + epoxyqueuosine(34) in tRNA |
|  | EC:2.7.14.1 | Protein arginine kinase | ATP + a [protein]-L-arginine <=> ADP + a [protein]-N(omega)-phospho-L-arginine |
|  | EC:2.8.4.3 | tRNA-2-methylthio-N(6)-dimethylallyladenosine synthase | N(6)-(3-methylbut-2-en-1-yl)-adenine(37) in tRNA + sulfur-(sulfur carrier) + 2 S-adenosyl-L-methionine + reduced electron acceptor <=> N(6)-(3-methylbut-2-en-1-yl)-2-(methylsulfanyl)adenine(37) in tRNA + S-adenosyl-L-homocysteine + (sulfur carrier) + L-methionine + 5'-deoxyadenosine + electron acceptor |
|  | EC:2.8.4.4 | [Ribosomal protein S12] (aspartate(89)-C(3))-methylthiotransferase | L-aspartate-[ribosomal protein S12] + sulfur-(sulfur carrier) + 2 S-adenosyl-L-methionine <=> 3-methylthio-L-aspartate-[ribosomal protein S12] + S-adenosyl-L-homocysteine + (sulfur carrier) + L-methionine + 5'-deoxyadenosine |
|  | EC:2.8.4.5 | tRNA (N(6)-L-threonylcarbamoyladenosine(37)-C(2))-methylthiotransferase | N(6)-L-threonylcarbamoyladenine(37) in tRNA + sulfur-(sulfur carrier) + 2 S-adenosyl-L-methionine + reduced electron acceptor <=> 2-methylthio-N(6)-L-threonylcarbamoyladenine(37) in tRNA + S-adenosyl-L-homocysteine + (sulfur carrier) + L-methionine + 5'-deoxyadenosine + electron acceptor |
|  | EC:3.1.13.1 | Exoribonuclease II | Exonucleolytic cleavage in the 3'- to 5'-direction to yield nucleoside 5'-phosphates |
|  | EC:3.3.1.1 | Adenosylhomocysteinase | S-adenosyl-L-homocysteine + H(2)O <=> L-homocysteine + adenosine |
|  | EC:3.4.25.2 | HslU--HslV peptidase | ATP-dependent cleavage of peptide bonds with broad specificity |
|  | EC:3.6.5.1 | Heterotrimeric G-protein GTPase | GTP + H(2)O <=> GDP + phosphate |
|  | EC:4.3.2.1 | Argininosuccinate lyase | 2-(N(omega)-L-arginino)succinate <=> fumarate + L-arginine |
|  | EC:4.3.2.2 | Adenylosuccinate lyase | N(6)-(1,2-dicarboxyethyl)AMP <=> fumarate + AMP  (S)-2-(5-amino-1-(5-phospho-D-ribosyl)imidazole-4-carboxamido)succinate <=> fumarate + 5-amino-1-(5-phospho-D-ribosyl)imidazole-4-carboxamide |
|  | EC:4.3.99.3 | 7-carboxy-7-deazaguanine synthase | 6-carboxy-5,6,7,8-tetrahydropterin <=> 7-carboxy-7-carbaguanine + NH(3) |
|  | EC:4.6.1.12 | 2-C-methyl-D-erythritol 2,4-cyclodiphosphate synthase | 2-phospho-4-(cytidine 5'-diphospho)-2-C-methyl-D-erythritol <=> 2-C-methyl-D-erythritol 2,4-cyclodiphosphate + CMP |
|  | EC:4.6.1.17 | Cyclic pyranopterin monophosphate synthase | (8S)-3',8-cyclo-7,8-dihydroguanosine 5'-triphosphate <=> cyclic pyranopterin phosphate + diphosphate |
|  | EC:5.1.99.6 | NAD(P)H-hydrate epimerase | (6R)-6-beta-hydroxy-1,4,5,6-tetrahydronicotinamide-adenine dinucleotide <=> (6S)-6-beta-hydroxy-1,4,5,6-tetrahydronicotinamide-adenine dinucleotide  (6R)-6-beta-hydroxy-1,4,5,6-tetrahydronicotinamide-adenine dinucleotide phosphate <=> (6S)-6-beta-hydroxy-1,4,5,6-tetrahydronicotinamide-adenine dinucleotide phosphate |
|  | EC:6.3.3.1 | Phosphoribosylformylglycinamidine cyclo-ligase | ATP + 2-(formamido)-N(1)-(5-phospho-D-ribosyl)acetamidine <=> ADP + phosphate + 5-amino-1-(5-phospho-D-ribosyl)imidazole |
|  | EC:6.3.3.3 | Dethiobiotin synthase | ATP + 7,8-diaminononanoate + CO(2) <=> ADP + phosphate + dethiobiotin |
|  | EC:6.5.1.2 | DNA ligase (NAD(+)) | NAD(+) + (deoxyribonucleotide)(n)-3'-hydroxyl + 5'-phospho-(deoxyribonucleotide)(m) <=> (deoxyribonucleotide)(n+m) + AMP + beta-nicotinamide D-nucleotide |
| **Accessory** | EC:1.1.2.4 | D-lactate dehydrogenase (cytochrome) | (R)-lactate + 2 ferricytochrome c <=> pyruvate + 2 ferrocytochrome c + 2 H(+) |
|  | EC:1.2.99.8 | Glyceraldehyde dehydrogenase (FAD-containing) | D-glyceraldehyde + H(2)O + acceptor <=> D-glycerate + reduced acceptor |
|  | EC:1.5.3.1 | Sarcosine oxidase | Sarcosine + H(2)O + O(2) <=> glycine + formaldehyde + H(2)O(2) |
|  | EC:1.5.99.14 | 6-hydroxypseudooxynicotine dehydrogenase | 1-(6-hydroxypyridin-3-yl)-4-(methylamino)butan-1-one + acceptor + H(2)O <=> 1-(2,6-dihydroxypyridin-3-yl)-4-(methylamino)butan-1-one + reduced acceptor |
|  | EC:1.6.99.1 | NADPH dehydrogenase | NADPH + acceptor <=> NADP(+) + reduced acceptor |
|  | EC:1.7.1.4 | Nitrite reductase (NAD(P)H) | Ammonia + 3 NAD(P)(+) + 2 H(2)O <=> nitrite + 3 NAD(P)H |
|  | EC:1.7.1.13 | PreQ(1) synthase | 7-aminomethyl-7-carbaguanine + 2 NADP(+) <=> 7-cyano-7-carbaguanine + 2 NADPH |
|  | EC:1.7.1.15 | Nitrite reductase (NADH) | Ammonia + 3 NAD(+) + 2 H(2)O <=> nitrite + 3 NADH |
|  | EC:1.7.99.4 | Nitrate reductase | Nitrite + acceptor <=> nitrate + reduced acceptor |
|  | EC:1.9.3.1 | Transferred to EC:[7.1.1.9](https://enzyme.expasy.org/EC/7.1.1.9) | |
|  | EC:1.14.11.17 | Taurine dioxygenase | Taurine + 2-oxoglutarate + O(2) <=> sulfite + aminoacetaldehyde + succinate + CO(2) |
|  | EC:1.14.12.17 | Nitric oxide dioxygenase | 2 nitric oxide + 2 O(2) + NAD(P)H <=> 2 nitrate + NAD(P)(+) + H(+) |
|  | EC:1.14.19.1 | Stearoyl-CoA 9-desaturase | Stearoyl-CoA + 2 ferrocytochrome b5 + O(2) + 2 H(+) <=> oleoyl-CoA + 2 ferricytochrome b5 + 2 H(2)O |
|  | EC:1.15.1.1 | Superoxide dismutase | 2 superoxide + 2 H(+) <=> O(2) + H(2)O(2) |
|  | EC:1.16.3.2 | Bacterial non-heme ferritin | 4 Fe(2+) + O(2) + 6 H(2)O <=> 4 (FeO(OH)) + 8 H(+) |
|  | EC:1.17.4.1 | Ribonucleoside-diphosphate reductase | 2'-deoxyribonucleoside diphosphate + thioredoxin disulfide + H(2)O <=> ribonucleoside diphosphate + thioredoxin |
|  | EC:1.18.1.2 | Ferredoxin--NADP(+) reductase | 2 reduced ferredoxin + NADP(+) + H(+) <=> 2 oxidized ferredoxin + NADPH |
|  | EC:2.7.3.9 | Phosphoenolpyruvate--protein phosphotransferase | Phosphoenolpyruvate + protein L-histidine <=> pyruvate + protein N(pi)-phospho-L-histidine |
|  | EC:2.7.9.3 | Selenide, water dikinase | ATP + selenide + H(2)O <=> AMP + selenophosphate + phosphate |
|  | EC:2.9.1.1 | L-seryl-tRNA(Sec) selenium transferase | L-seryl-tRNA(Sec) + selenophosphate <=> L-selenocysteinyl-tRNA(Sec) + phosphate |
|  | EC:3.1.11.6 | Exodeoxyribonuclease VII | Exonucleolytic cleavage in either 5'- to 3'- or 3'- to 5'-direction to yield nucleoside 5'-phosphates |
|  | EC:3.4.14.13 | Gamma-D-glutamyl-L-lysine dipeptidyl-peptidase | The enzyme releases L-Ala-gamma-D-Glu dipeptides from cell wall peptides via cleavage of an L-Ala-gamma-D-Glu-\|-L-Lys bond |
|  | EC:3.4.16.4 | Serine-type D-Ala-D-Ala carboxypeptidase | Preferential cleavage: (Ac)(2)-L-Lys-D-Ala-\|-D-Ala. Also transpeptidation of peptidyl-alanyl moieties that are N-acyl substituents of D-alanine |
|  | EC:3.4.19.3 | Pyroglutamyl-peptidase I | Release of an N-terminal pyroglutamyl group from a polypeptide, the second amino acid generally not being Pro |
|  | EC:3.5.99.2 | Aminopyrimidine aminohydrolase | 4-amino-5-aminomethyl-2-methylpyrimidine + H(2)O <=> 4-amino-5-hydroxymethyl-2-methylpyrimidine + ammonia  Thiamine + H(2)O <=> 4-amino-5-hydroxymethyl-2-methylpyrimidine + 5-(2-hydroxyethyl)-4-methylthiazole |
|  | EC:3.5.99.6 | Glucosamine-6-phosphate deaminase | Alpha-D-glucosamine 6-phosphate + H(2)O <=> D-fructose 6-phosphate + NH(3) |
|  | EC:3.7.1.2 | Fumarylacetoacetase | 4-fumarylacetoacetate + H(2)O <=> acetoacetate + fumarate |
|  | EC:3.7.1.9 | 2-hydroxymuconate-6-semialdehyde hydrolase | 2-hydroxymuconate-6-semialdehyde + H(2)O <=> formate + 2-oxopent-4-enoate |
|  | EC:3.7.1.22 | 3D-(3,5/4)-trihydroxycyclohexane-1,2-dione acylhydrolase (decyclizing) | 3D-3,5/4-trihydroxycyclohexa-1,2-dione + H(2)O <=> 5-deoxy-D-glucuronate |
|  | EC:3.8.1.2 | (S)-2-haloacid dehalogenase | (S)-2-haloacid + H(2)O <=> (R)-2-hydroxyacid + halide |
|  | EC:3.11.1.1 | Phosphonoacetaldehyde hydrolase | Phosphonoacetaldehyde + H(2)O <=> acetaldehyde + phosphate |
|  | EC:5.3.3.2 | Isopentenyl-diphosphate Delta-isomerase | Isopentenyl diphosphate <=> dimethylallyl diphosphate |
| **Unique** | EC:1.14.13.131 | Dissimilatory dimethyl-sulfide monooxygenase | Dimethyl sulfide + O(2) + NADH <=> methanethiol + formaldehyde + NAD(+) + H(2)O |
|  | EC:1.14.13.236 | Toluene 4-monooxygenase | Toluene + NADH + O(2) <=> 4-methylphenol + NAD(+) + H(2)O |
|  | EC:1.18.6.1 | Nitrogenase | 8 reduced ferredoxin + 8 H(+) + N(2) + 16 ATP + 16 H(2)O <=> 8 oxidized ferredoxin + H(2) + 2 NH(3) + 16 ADP + 16 phosphate |
|  | EC:1.97.1.4 | [Formate-C-acetyltransferase]-activating enzyme | S-adenosyl-L-methionine + dihydroflavodoxin + [formate C-acetyltransferase]-glycine <=> 5'-deoxyadenosine + L-methionine + flavodoxin semiquinone + [formate C-acetyltransferase]-glycin-2-yl radical |
|  | EC:5.3.99.11 | 2-keto-myo-inositol isomerase | 2,4,6/3,5-pentahydroxycyclohexanone <=> 2D-2,3,5/4,6-pentahydroxycyclohexanone |
|  | EC:3.1.4.52 | Cyclic-guanylate-specific phosphodiesterase | Cyclic di-3',5'-guanylate + H(2)O <=> 5'-phosphoguanylyl(3'->5')guanosine |

**Table S3: Distribution of regions with biosynthetic potential in *Aneurinibacillus***

| **Organism** | **Region** | **Type** | **From** | **To** | **Most similar known cluster** | **MIBiG** | **Similarity**  **(%)** |
| --- | --- | --- | --- | --- | --- | --- | --- |
| ***A. aneurinilyticus* ATCC 12856^T^** | 31.1 | NRPS | 1 | 45536 | Minutissamide A / Minutissamide C / Minutissamide D | BGC0001952 | 15 |
|  | 79.1 | NRPS | 1 | 17929 | - | - | - |
|  | 87.1 | Betalactone | 1 | 22678 | - | - | - |
|  | 93.1 | Bacteriocin | 1 | 14290 | - | - | - |
|  | 100.1 | [T3PKS](../../../../../../../C:/Users/majid/Downloads/_blank) | 1 | 41474 | - | - | - |
|  | 109.1 | NRPS | 1 | 23101 | Octapeptin | BGC0001715 | 11 |
|  | 225.1 | NRPS | 1 | 48733 | - | - | - |
|  | 262.1 | [Terpene](../../../../../../../C:/Users/majid/Downloads/_blank) | 1 | 5231 | - | - | - |
|  | 266.1 | Bacteriocin | 1 | 14305 | - | - | - |
|  | 269.1 | Bacteriocin | 1 | 14299 | - | - | - |
| ***A. danicus* NBRC 102444^T^** | 13.1 | Siderophore | 6835 | 23576 | - | - | - |
|  | 20.1 | Betalactone | 2690 | 32158 | - | - | - |
|  | 46.1 | Bacteriocin | 5864 | 16718 | - | - | - |
|  | 54.1 | [T3PKS](../../../../../../../C:/Users/majid/Downloads/_blank) | 14764 | 42210 | - | - | - |
|  | 164.1 | [Terpene](../../../../../../../C:/Users/majid/Downloads/_blank) | 5915 | 26702 | - | - | - |
|  | 175.1 | LAP bacteriocin | 1 | 14369 | - | - | - |
| ***A. migulanus* DSM 2895^T^** | 15.1 | [Terpene](../../../../../../../C:/Users/majid/Downloads/_blank) | 93,143 | 113,961 | - | - | - |
|  | 17.1 | Siderophore | 111,719 | 128,417 | - | - | - |
|  | 18.1 | Phosphonate | 264,342 | 305,946 | - | - | - |
|  | 18.2 | Bacteriocin | 2,136,406 | 2,147,260 | - | - | - |
|  | 18.3 | NRPS | 2,296,298 | 2,343,464 | - | - | - |
|  | 18.4 | [T3PKS](../../../../../../../C:/Users/majid/Downloads/_blank) | 2,727,750 | 2,768,808 | [Molybdenum cofactor](https://mibig.secondarymetabolites.org/go/BGC0000916/1) | BGC0000916 | 11 |
|  | 18.5 | Terpene | 3,066,995 | 3,088,914 | - | - | - |
|  | 18.6 | NRPS | 3,284,920 | 3,341,639 | [Tyrocidine](https://mibig.secondarymetabolites.org/go/BGC0000452/1) | BGC0000452 | 18 |
|  | 18.7 | Lassopeptide | 4,073,522 | 4,097,668 | [Paeninodin](https://mibig.secondarymetabolites.org/go/BGC0001356/1) | BGC0001356 | 80 |
|  | 18.8 | Betalactone | 4,821,336 | 4,850,763 | - | - | - |
| ***A. soli* CB4^T^** | 1 | [T3PKS](../../../../../../../C:/Users/majid/Downloads/_blank) | 2,452,882 | 2,493,946 | [Molybdenum cofactor](https://mibig.secondarymetabolites.org/go/BGC0000916/1)/ Other | BGC0000916 | 17 |
|  | 2 | [NRPS-like](../../../../../../../C:/Users/majid/Downloads/_blank),[terpene](../../../../../../../C:/Users/majid/Downloads/_blank) | 2,614,904 | 2,658,254 | [Pimaricin](https://mibig.secondarymetabolites.org/go/BGC0000125/1)/Polyketide | BGC0000125 | 11 |
|  | 3 | [Terpene](../../../../../../../C:/Users/majid/Downloads/_blank) | 2,732,430 | 2,753,254 | - | - | - |
|  | 4 | [NRPS](../../../../../../../C:/Users/majid/Downloads/_blank) | 2,838,391 | 2,882,137 | [Surfactin](https://mibig.secondarymetabolites.org/go/BGC0000433/1)/NRP:Lipopeptide | BGC0000433 | 8 |
| ***Aneurinibacillus* sp. UBA3580** | 38.1 | Siderophore | 5,466 | 18,190 | - | - | - |
|  | 62.1 | [T3PKS](../../../../../../../C:/Users/majid/Downloads/_blank) | 1 | 27,511 | - | - | - |
|  | 81.1 | [Terpene](../../../../../../../C:/Users/majid/Downloads/_blank) | 36,923 | 53,246 | - | - | - |
|  | 98.1 | LAP/ bacteriocin | 1 | 22,205 | - | - | - |
| ***Aneurinibacillus* sp. XH2** | 1 | [Bacteriocin](../../../../../../../C:/Users/majid/Downloads/_blank) | 4,580 | 13,845 | - | - | - |
|  | 2 | [Siderophore](../../../../../../../C:/Users/majid/Downloads/_blank) | 507,169 | 528,261 | [Staphylobactin](https://mibig.secondarymetabolites.org/go/BGC0000943/1)/ Other | BGC0000943 | 18 |
|  | 3 | Arylpolyene | 922,842 | 963,975 | - | - | - |
|  | 4 | Hserlactone | 1,136,603 | 1,157,361 | Mycosubtilin/  NRP + Polyketide | BGC0001103 | 20 |
|  | 5 | [LAP](../../../../../../../C:/Users/majid/Downloads/_blank)/[bacteriocin](../../../../../../../C:/Users/majid/Downloads/_blank) | 2,298,825 | 2,322,382 | - | - | - |
|  | 6 | [Betalactone](../../../../../../../C:/Users/majid/Downloads/_blank) | 2,708,133 | 2,737,525 | - | - | - |
|  | 7 | Sactipeptide | 3,177,879 | 3,199,240 | - | - | - |
|  | 8 | Lanthipeptide | 3,224,594 | 3,251,065 | [Geobacillin I](https://mibig.secondarymetabolites.org/go/BGC0000515/1)/ RiPP:Lanthipeptide | BGC0000515 | 90 |
| ***A. terranovensis* DSM 18919^T^** | 4.1 | T3PKS | 48,631 | 89,686 | - | - | - |
|  | 22.1 | [NRPS](../../../../../../../C:/Users/majid/Downloads/_blank) | 18,083 | 61,909 | [Surfactin](https://mibig.secondarymetabolites.org/go/BGC0000433/1) | BGC0000433 | 8 |
| ***A. thermoaerophilus* L 420-91^T^** | 16.1 | Hserlactone | 679 | 21,455 | [Mycosubtilin](https://mibig.secondarymetabolites.org/go/BGC0001103/1)  NRP + Polyketide | BGC0001103 | 20 |
|  | 18.1 | \|  \|  \|  \|  \| \| --- \| --- \| --- \| --- \|   Lanthipeptide | 167,917 | 183,936 | GeobacillinI/RiPP:Lanthipeptide | BGC0000515 | 50 |
|  | 31.1 | [Sactipeptide](../../../../../../../C:/Users/majid/Downloads/_blank) | 2433 | 23794 | - | - | - |
|  | 49.1 | Betalactone | 83,485 | 112,877 | - | - | - |
|  | 51.1 | [Arylpolyene](../../../../../../../C:/Users/majid/Downloads/_blank) | 53,126 | 94,259 | - | - | - |
|  | 52.1 | [LAP](../../../../../../../C:/Users/majid/Downloads/_blank)/[bacteriocin](../../../../../../../C:/Users/majid/Downloads/_blank) | 38,680 | 56,469 | - | - | - |
|  | 60.1 | [Siderophore](../../../../../../../C:/Users/majid/Downloads/_blank) | 1 | 16,436 | Staphylobactin/ others | BGC0000943 | 18 |
|  | 95.1 | [Bacteriocin](../../../../../../../C:/Users/majid/Downloads/_blank) | 3,318 | 14,166 | - | - | - |
| ***A. tyrosinisolvens* LL-002^T^** | 1.1 | [Lanthipeptide](../../../../../../../C:/Users/majid/Downloads/_blank) | \| 164,848 \|  \| \| --- \| --- \| | 188,036 | - | - | - |
|  | 4.1 | [Bacteriocin](../../../../../../../C:/Users/majid/Downloads/_blank) | \| 173,536 \|  \| \| --- \| --- \| | 184,384 | - | - | - |
|  | 6.1 | NRPS | \| 1 \|  \| \| --- \| --- \| | 91,624 | Lichenysin | BGC0000381 | 35 |
|  | 11.1 | Terpene | \| 11,5414 \|  \| \| --- \| --- \| | 136,229 | - | - | - |
|  | 13.1 | T3PKS | \| 21,891 \|  \| \| --- \| --- \| | 62,946 | [Molybdenum cofactor](https://mibig.secondarymetabolites.org/go/BGC0000916/1) | BGC0000916 | 11 |
|  | 22.1 | Betalactone | \| 43,094 \|  \| \| --- \| --- \| | 72,760 | - | - | - |
|  | 109.1 | [Bacteriocin](../../../../../../../C:/Users/majid/Downloads/_blank) | \| 1 \|  \| \| --- \| --- \| | 1,392 | - | - | - |

**Table S4: Distribution of CAZymes and their various families in *Aneurinibacillus*. Values in parenthesis (column 3-8) represent different types of CAZy families.**

| **Genome Name** | **No. of genes (%)** | **No. of GH** | **No. of GT** | **No. of CE** | **No. of PL** | **No. of CBM** | **No. of AA** |
| --- | --- | --- | --- | --- | --- | --- | --- |
| *A. aneurinilyticus* ATCC 12856^T^ | 61 (0.98) | 14 (6) | 21 (7) | 20 (6) | 0 (0) | 1 (1) | 7 (3) |
| *A. danicus* NBRC 102444^T^ | 66 (1.34) | 12 (8) | 36 (10) | 13 (5) | 1 (1) | 2 (2) | 4 (1) |
| *A. migulanus* DSM 2895^T^ | 54 (0.95) | 6 (4) | 20 (6) | 21 (6) | 0 (0) | 0 (0) | 7 (3) |
| *A. soli* CB4^T^ | 63 (1.58) | 6 (4) | 25 (5) | 20 (4) | 0 (0) | 1 (1) | 4 (2) |
| *Aneurinibacillus* sp. UBA3580 | 54 (1.36) | 10 (8) | 25 (7) | 11 (4) | 1 (1) | 1 (1) | 6 (3) |
| *Aneurinibacillus* sp. XH2 | 47 (1.37) | 5 (4) | 23 (6) | 12 (4) | 0 (0) | 1 (1) | 5 (2) |
| *A. terranovensis* DSM 18919^T^ | 68 (1.72) | 15 (7) | 26 (9) | 22 (5) | 1 (1) | 4 (3) | 3 (2) |
| *A. thermoaerophilus* L 420-91^T^ | 49 (1.39) | 5 (4) | 25 (6) | 12 (4) | 0 (0) | 1 (1) | 5 (2) |
| *A. tyrosinisolvens* LL-002^T^ | 89 (1.66) | 26 (10) | 31 (8) | 22 (4) | 0 (0) | 3 (2) | 10 (3) |

**Table S5: List of all CAZy genes identified in this work. Some genes have more than one CAZy domain.**

| **Protein Accession** | **CAZy Family** | **Signal Peptide** |
| --- | --- | --- |
| AMA71549.1 | CE4 | NO |
| AMA71579.1 | GT51 | NO |
| AMA71670.1 | GH109 | NO |
| AMA71688.1 | GT26 | NO |
| AMA71697.1 | GT4 | NO |
| AMA71702.1 | GT4 | NO |
| AMA71703.1 | GT4 | NO |
| AMA71704.1 | GT4 | NO |
| AMA71881.1 | AA4 | NO |
| AMA72036.1 | GT4 | NO |
| AMA72262.1 | CE4 | SP(Sec/SPI) |
| AMA72286.1 | CE1 | NO |
| AMA72387.1 | CE4 | SP(Sec/SPI) |
| AMA72455.1 | GT2 | NO |
| AMA72571.1 | GT28 | NO |
| AMA72627.1 | SLH | SP(Sec/SPI) |
| AMA72714.1 | CE4 | SP(Sec/SPI) |
| AMA72810.1 | GT2 | NO |
| AMA72957.1 | GT4 | NO |
| AMA72959.1 | GH73 | SP(Sec/SPI) |
| AMA72972.1 | CE1 | NO |
| AMA73038.1 | GT4 | NO |
| AMA73039.1 | GT2 | NO |
| AMA73122.1 | AA4 | NO |
| AMA73233.1 | CE14 | NO |
| AMA73256.1 | GT2 | NO |
| AMA73257.1 | GT2 | NO |
| AMA73285.1 | GT94 | NO |
| AMA73291.1 | CBM50 | NO |
| AMA73302.1 | GH18 | NO |
| AMA73363.1 | GT28 | NO |
| AMA73396.1 | CE4 | SP(Sec/SPI) |
| AMA73544.1 | GT4 | NO |
| AMA73545.1 | CE14 | NO |
| AMA73637.1 | CE9 | NO |
| AMA73720.1 | GH23 | NO |
| AMA73730.1 | CE4 | NO |
| AMA73824.1 | GT51 | NO |
| AMA73878.1 | GT51 | LIPO(Sec/SPII) |
| AMA73955.1 | GH23 | NO |
| AMA74193.1 | AA4 | NO |
| AMA74270.1 | GT2 | NO |
| AMA74390.1 | CE4 | SP(Sec/SPI) |
| AMA74430.1 | AA3 | NO |
| AMA74674.1 | GT4 | NO |
| AMA74751.1 | GT2 | NO |
| AMA74794.1 | AA4 | NO |
| BAU25966.1 | CE4 | SP(Sec/SPI) |
| BAU26063.1 | CE4 | SP(Sec/SPI) |
| BAU26313.1 | SLH | SP(Sec/SPI) |
| BAU26314.1 | SLH | SP(Sec/SPI) |
| BAU26393.1 | CE4 | NO |
| BAU26403.1 | CE4 | SP(Sec/SPI) |
| BAU26418.1 | GT51 | NO |
| BAU26497.1 | GT26 | NO |
| BAU26503.1 | GT2 | NO |
| BAU26511.1 | GT2 | NO |
| BAU26521.1 | GT2 | NO |
| BAU26536.1 | CBM54 | SP(Sec/SPI) |
|  | SLH |  |
|  | SLH |  |
| BAU26670.1 | CE4 | LIPO(Sec/SPII) |
| BAU26690.1 | AA4 | NO |
| BAU26732.1 | SLH | LIPO(Sec/SPII) |
|  | SLH |  |
| BAU26925.1 | GT4 | NO |
| BAU27076.1 | CE4 | NO |
|  | GH18 |  |
|  | GT2 |  |
| BAU27082.1 | GT28 | NO |
| BAU27083.1 | CE4 | NO |
| BAU27112.1 | cohesin | SP(Sec/SPI) |
|  | SLH |  |
|  | SLH |  |
| BAU27134.1 | CE1 | NO |
| BAU27394.1 | AA4 | NO |
| BAU27490.1 | GT28 | NO |
| BAU27721.1 | CE4 | SP(Sec/SPI) |
| BAU27769.1 | SLH | SP(Sec/SPI) |
| BAU27844.1 | AA6 | NO |
| BAU27893.1 | SLH | SP(Sec/SPI) |
| BAU28028.1 | GH18 | SP(Sec/SPI) |
| BAU28048.1 | CE4 | LIPO(Sec/SPII) |
| BAU28087.1 | GT4 | NO |
| BAU28092.1 | GT4 | NO |
| BAU28094.1 | GT4 | NO |
| BAU28098.1 | GT4 | NO |
| BAU28299.1 | CE14 | NO |
| BAU28300.1 | GT4 | NO |
| BAU28316.1 | GT51 | NO |
| BAU28438.1 | CE4 | SP(Sec/SPI) |
| BAU28550.1 | GH126 | NO |
| BAU28551.1 | GT2 | NO |
| BAU28616.1 | GT2 | NO |
| BAU28702.1 | CE4 | SP(Sec/SPI) |
| BAU28713.1 | CE4 | SP(Sec/SPI) |
| BAU28862.1 | GT4 | NO |
| BAU28863.1 | CE14 | NO |
| BAU29082.1 | GH2 | NO |
| BAU29083.1 | CE9 | NO |
| BAU29144.1 | GT2 | NO |
| BAU29161.1 | CE4 | SP(Sec/SPI) |
| BAU29188.1 | GH23 | NO |
| BAU29201.1 | CE4 | SP(Sec/SPI) |
| BAU29314.1 | GT51 | SP(Sec/SPI) |
| BAU29393.1 | GT51 | LIPO(Sec/SPII) |
| BAU29430.1 | GT4 | NO |
| BAU29440.1 | CE4 | SP(Sec/SPI) |
| BAU29445.1 | SLH | SP(Sec/SPI) |
| BAU29510.1 | GH23 | NO |
| BAU29707.1 | GT2 | NO |
| BAU29709.1 | CE4 | NO |
| BAU29715.1 | GT4 | NO |
| BAU29800.1 | SLH | SP(Sec/SPI) |
| BAU29821.1 | SLH | SP(Sec/SPI) |
|  | SLH |  |
| BAU29827.1 | AA4 | NO |
| BAU29913.1 | GT2 | NO |
| ERI05052.1 | GT4 | NO |
| ERI05053.1 | GT2 | NO |
| ERI05054.1 | GT2 | NO |
| ERI05164.1 | GT2 | NO |
| ERI05460.1 | CE4 | SP(Sec/SPI) |
| ERI05567.1 | CE4 | SP(Sec/SPI) |
| ERI05910.1 | GT2 | NO |
| ERI05964.1 | CE9 | NO |
| ERI05965.1 | GH2 | NO |
| ERI06018.1 | GH23 | NO |
| ERI06444.1 | GH25 | NO |
| ERI06449.1 | GT51 | NO |
| ERI06450.1 | GH25 | NO |
| ERI06743.1 | CE4 | SP(Sec/SPI) |
| ERI06792.1 | AA3 | NO |
| ERI06800.1 | GT4 | NO |
| ERI06894.1 | GT28 | NO |
| ERI07043.1 | GT4 | NO |
| ERI07049.1 | GH109 | NO |
| ERI07109.1 | GT89 | NO |
| ERI07115.1 | GT26 | NO |
| ERI07173.1 | CE4 | NO |
| ERI07233.1 | GH23 | NO |
| ERI07324.1 | GH23 | NO |
| ERI07615.1 | AA4 | NO |
| ERI07811.1 | CE4 | NO |
| ERI07925.1 | CE1 | NO |
| ERI08228.1 | GT51 | LIPO(Sec/SPII) |
| ERI08247.1 | GH18 | LIPO(Sec/SPII) |
| ERI08328.1 | GT4 | NO |
| ERI08329.1 | CE14 | NO |
| ERI08449.1 | CBM54 | NO |
| ERI08478.1 | GT51 | NO |
| ERI08624.1 | GH23 | NO |
| ERI08646.1 | CE4 | NO |
| ERI08647.1 | GT28 | NO |
| ERI08652.1 | CE4 | NO |
|  | GH18 |  |
|  | GT2 |  |
| ERI08707.1 | CE4 | LIPO(Sec/SPII) |
| ERI08818.1 | CE4 | NO |
| ERI08901.1 | GT1 | NO |
| ERI09064.1 | AA6 | NO |
| ERI09118.1 | GH25 | NO |
| ERI09129.1 | CE4 | NO |
| ERI09295.1 | CE4 | SP(Sec/SPI) |
| ERI09370.1 | CE4 | LIPO(Sec/SPII) |
| ERI09380.1 | AA4 | NO |
| ERI09546.1 | AA6 | NO |
| ERI09578.1 | GT4 | NO |
| ERI10017.1 | CE4 | SP(Sec/SPI) |
| ERI10171.1 | GT51 | NO |
| ERI10297.1 | GT4 | NO |
| ERI10367.1 | CE7 | NO |
| ERI10595.1 | AA4 | NO |
| ERI10646.1 | CE10 | NO |
| ERI10678.1 | AA4 | NO |
| ERI10778.1 | CE1 | NO |
| ERI10837.1 | GH73 | LIPO(Sec/SPII) |
| ERI10876.1 | GH25 | NO |
| ERI10969.1 | CE1 | NO |
| ERI11441.1 | GT28 | NO |
| ERI11634.1 | GH73 | SP(Sec/SPI) |
| fig\|1946041.3.peg.177 | GH23 | SP(Sec/SPI) |
| fig\|1946041.3.peg.197 | CE4 | SP(Sec/SPI) |
| fig\|1946041.3.peg.260 | CE4 | SP(Sec/SPI) |
| fig\|1946041.3.peg.330 | CE14 | NO |
| fig\|1946041.3.peg.504 | GT4 | NO |
| fig\|1946041.3.peg.526 | GT2 | NO |
| fig\|1946041.3.peg.553 | CE4 | NO |
| fig\|1946041.3.peg.625 | GT51 | LIPO(Sec/SPII) |
| fig\|1946041.3.peg.710 | GT2 | NO |
| fig\|1946041.3.peg.711 | GT2 | NO |
| fig\|1946041.3.peg.712 | GT4 | NO |
| fig\|1946041.3.peg.713 | GT4 | NO |
| fig\|1946041.3.peg.714 | GT2 | NO |
| fig\|1946041.3.peg.720 | GT4 | NO |
| fig\|1946041.3.peg.758 | GH18 | LIPO(Sec/SPII) |
| fig\|1946041.3.peg.770 | CE4 | LIPO(Sec/SPII) |
| fig\|1946041.3.peg.791 | GT2 | NO |
| fig\|1946041.3.peg.829 | GT51 | NO |
| fig\|1946041.3.peg.860 | PL17 | NO |
| fig\|1946041.3.peg.1037 | CE4 | SP(Sec/SPI) |
| fig\|1946041.3.peg.1175 | CE4 | SP(Sec/SPI) |
| fig\|1946041.3.peg.1490 | GH2 | NO |
| fig\|1946041.3.peg.1493 | CE9 | NO |
| fig\|1946041.3.peg.1679 | GH109 | NO |
| fig\|1946041.3.peg.1686 | GT4 | NO |
| fig\|1946041.3.peg.1701 | GT26 | NO |
| fig\|1946041.3.peg.1726 | GT4 | NO |
| fig\|1946041.3.peg.1810 | CBM48 | NO |
|  | GH13 |  |
| fig\|1946041.3.peg.1814 | GT5 | NO |
| fig\|1946041.3.peg.1815 | GT35 | NO |
| fig\|1946041.3.peg.1816 | GH13 | NO |
| fig\|1946041.3.peg.2042 | CE1 | NO |
| fig\|1946041.3.peg.2122 | GT2 | NO |
| fig\|1946041.3.peg.2160 | GT4 | NO |
| fig\|1946041.3.peg.2183 | AA4 | NO |
| fig\|1946041.3.peg.2368 | CE4 | SP(Sec/SPI) |
| fig\|1946041.3.peg.2384 | GH23 | NO |
| fig\|1946041.3.peg.2464 | GT28 | NO |
| fig\|1946041.3.peg.2625 | GT4 | NO |
| fig\|1946041.3.peg.2750 | GT28 | NO |
| fig\|1946041.3.peg.2853 | AA6 | NO |
| fig\|1946041.3.peg.2855 | AA7 | NO |
| fig\|1946041.3.peg.2887 | GH73 | NO |
| fig\|1946041.3.peg.2940 | SLH | NO |
| fig\|1946041.3.peg.3179 | AA4 | NO |
| fig\|1946041.3.peg.3273 | AA4 | NO |
| fig\|1946041.3.peg.3275 | GT4 | NO |
| fig\|1946041.3.peg.3396 | GT2 | NO |
| fig\|1946041.3.peg.3459 | AA4 | NO |
| fig\|1946041.3.peg.3602 | GH88 | NO |
| fig\|1946041.3.peg.3641 | GT51 | NO |
| fig\|1946041.3.peg.3705 | CE4 | SP(Sec/SPI) |
| fig\|1946041.3.peg.3707 | GT4 | NO |
| fig\|1946041.3.peg.3929 | GH31 | NO |
| fig\|267746.3.peg.102\|ADA01nite_00920 | GH23 | SP(Sec/SPI) |
| fig\|267746.3.peg.122\|ADA01nite_01100 | CE4 | SP(Sec/SPI) |
| fig\|267746.3.peg.217\|ADA01nite_01980 | GT28 | NO |
| fig\|267746.3.peg.345\|ADA01nite_03140 | AA4 | NO |
| fig\|267746.3.peg.706\|ADA01nite_06400 | GT51 | NO |
| fig\|267746.3.peg.992\|ADA01nite_09000 | GT81 | NO |
| fig\|267746.3.peg.1052\|ADA01nite_09540 | CE9 | NO |
| fig\|267746.3.peg.1053\|ADA01nite_09550 | GH2 | NO |
| fig\|267746.3.peg.1111\|ADA01nite_10080 | GT2 | NO |
| fig\|267746.3.peg.1133\|ADA01nite_10290 | GT4 | NO |
| fig\|267746.3.peg.1213\|ADA01nite_11010 | CE4 | SP(Sec/SPI) |
| fig\|267746.3.peg.1378\|ADA01nite_12490 | GT35 | NO |
| fig\|267746.3.peg.1379\|ADA01nite_12500 | GT5 | NO |
| fig\|267746.3.peg.1382\|ADA01nite_12530 | CBM48 | NO |
|  | GH13 |  |
| fig\|267746.3.peg.1449\|ADA01nite_13200 | CBM50 | NO |
| fig\|267746.3.peg.1564\|ADA01nite_14250 | AA4 | NO |
| fig\|267746.3.peg.1634\|ADA01nite_14870 | GH88 | NO |
| fig\|267746.3.peg.1701\|ADA01nite_15490 | GT4 | NO |
| fig\|267746.3.peg.1703\|ADA01nite_15510 | CE4 | SP(Sec/SPI) |
| fig\|267746.3.peg.1794\|ADA01nite_16340 | GT4 | NO |
| fig\|267746.3.peg.1796\|ADA01nite_16360 | GH109 | NO |
| fig\|267746.3.peg.1801\|ADA01nite_16410 | GT4 | NO |
| fig\|267746.3.peg.1884\|ADA01nite_17230 | CE14 | NO |
| fig\|267746.3.peg.1885\|ADA01nite_17240 | GT4 | NO |
| fig\|267746.3.peg.2266\|ADA01nite_20810 | CE1 | NO |
| fig\|267746.3.peg.2490\|ADA01nite_22870 | CE4 | SP(Sec/SPI) |
| fig\|267746.3.peg.2498\|ADA01nite_22940 | GT28 | NO |
| fig\|267746.3.peg.2604\|ADA01nite_23810 | GH31 | NO |
| fig\|267746.3.peg.2632\|ADA01nite_24060 | GT51 | LIPO(Sec/SPII) |
| fig\|267746.3.peg.2677\|ADA01nite_24510 | GT2 | NO |
| fig\|267746.3.peg.2725\|ADA01nite_24930 | GT2 | NO |
| fig\|267746.3.peg.2834\|ADA01nite_25950 | GT26 | NO |
| fig\|267746.3.peg.2850\|ADA01nite_26080 | GT2 | NO |
| fig\|267746.3.peg.2851\|ADA01nite_26090 | GT2 | NO |
|  | GT2 |  |
| fig\|267746.3.peg.2852\|ADA01nite_26100 | GT2 | NO |
| fig\|267746.3.peg.2863\|ADA01nite_26210 | GT2 | NO |
| fig\|267746.3.peg.2916\|ADA01nite_26690 | GT4 | NO |
| fig\|267746.3.peg.3028\|ADA01nite_27660 | GT51 | NO |
| fig\|267746.3.peg.3131\|ADA01nite_28500 | CE4 | SP(Sec/SPI) |
| fig\|267746.3.peg.3145\|ADA01nite_28640 | GH23 | NO |
| fig\|267746.3.peg.3377\|ADA01nite_30670 | CE1 | NO |
| fig\|267746.3.peg.3468\|ADA01nite_31450 | PL17 | NO |
| fig\|267746.3.peg.3507\|ADA01nite_31780 | AA4 | NO |
| fig\|267746.3.peg.3705\|ADA01nite_33390 | CE4 | NO |
| fig\|267746.3.peg.3708\|ADA01nite_33420 | CE4 | LIPO(Sec/SPII) |
| fig\|267746.3.peg.3727\|ADA01nite_33610 | GT2 | NO |
| fig\|267746.3.peg.3852\|ADA01nite_34650 | GT4 | NO |
| fig\|267746.3.peg.3861\|ADA01nite_34710 | GH109 | NO |
| fig\|267746.3.peg.4034\|ADA01nite_36280 | GH73 | NO |
| fig\|267746.3.peg.4072\|ADA01nite_36620 | GT4 | NO |
| fig\|267746.3.peg.4133\|ADA01nite_37140 | GH18 | LIPO(Sec/SPII) |
| fig\|267746.3.peg.4144\|ADA01nite_37250 | GT4 | NO |
| fig\|267746.3.peg.4145\|ADA01nite_37260 | GT2 | NO |
| fig\|267746.3.peg.4152 | GT27 | NO |
| fig\|267746.3.peg.4205 | GH13 | NO |
| fig\|267746.3.peg.4311\|ADA01nite_38740 | GT4 | NO |
| fig\|267746.3.peg.4319\|ADA01nite_38820 | CE4 | SP(Sec/SPI) |
| fig\|267746.3.peg.4402\|ADA01nite_39570 | GH18 | LIPO(Sec/SPII) |
| fig\|267746.3.peg.4412\|ADA01nite_39640 | CE10 | NO |
| fig\|267746.3.peg.4538\|ADA01nite_40760 | AA4 | NO |
| fig\|267746.3.peg.4665\|ADA01nite_41790 | GT4 | NO |
| fig\|267746.3.peg.4667\|ADA01nite_41810 | GT4 | NO |
| fig\|267746.3.peg.4678\|ADA01nite_41860 | GT2 | NO |
| fig\|267746.3.peg.4679\|ADA01nite_41870 | GT32 | NO |
| fig\|267746.3.peg.4680\|ADA01nite_41880 | GT2 | NO |
| fig\|267746.3.peg.4725\|ADA01nite_42260 | GT28 | NO |
| KON84106.1 | AA4 | NO |
| KON90709.1 | CE4 | SP(Sec/SPI) |
| KON90751.1 | AA3 | NO |
| KON90757.1 | GT4 | NO |
| KON93227.1 | AA6 | NO |
| KON94463.1 | GT51 | NO |
| KON94542.1 | GH109 | NO |
| KON94547.1 | GT4 | NO |
| KON94559.1 | GT26 | NO |
| KON94563.1 | GT89 | NO |
| KON94617.1 | GT4 | NO |
| KON94771.1 | AA4 | NO |
| KON95001.1 | GT4 | NO |
| KON95274.1 | CE4 | NO |
| KON95332.1 | CE1 | NO |
| KON95513.1 | GT2 | NO |
| KON95665.1 | GT28 | NO |
| KON95813.1 | CE4 | SP(Sec/SPI) |
| KON95953.1 | CE4 | NO |
| KON96038.1 | CE4 | SP(Sec/SPI) |
| KON96109.1 | CE7 | NO |
| KON96158.1 | CE4 | SP(Sec/SPI) |
| KON96242.1 | GH18 | SP(Sec/SPI) |
| KON96441.1 | AA4 | NO |
| KON96442.1 | AA4 | NO |
| KON96530.1 | GT28 | NO |
| KON96698.1 | CE4 | NO |
| KON96834.1 | GT4 | NO |
| KON96892.1 | GT28 | NO |
| KON96994.1 | CE1 | NO |
| KON97178.1 | CE1 | NO |
| KON97392.1 | GT51 | NO |
| KON97603.1 | GT4 | NO |
| KON97617.1 | GT2 | NO |
| KON97675.1 | GH109 | NO |
| KON97966.1 | GT4 | NO |
| KON98085.1 | GH2 | NO |
| KON98086.1 | CE9 | NO |
| KON98131.1 | GT2 | NO |
| KON98312.1 | CE4 | NO |
| KON98398.1 | CE4 | NO |
| KON98403.1 | AA4 | NO |
| KON98408.1 | CE10 | NO |
| KON98590.1 | CE1 | NO |
| KON98659.1 | CE4 | NO |
| KON98691.1 | CE14 | NO |
| KON98716.1 | CE4 | NO |
| KON99191.1 | GT51 | LIPO(Sec/SPII) |
| KON99229.1 | GT4 | NO |
| KON99313.1 | GH23 | SP(Sec/SPI) |
| KON99324.1 | GH109 | NO |
| KON99328.1 | GT2 | NO |
| KON99437.1 | CE4 | NO |
| KON99451.1 | CE4 | NO |
| WP_027414494.1 | GT28 | NO |
| WP_027414652.1 | CE4 | SP(Sec/SPI) |
| WP_027414771.1 | GH23 | NO |
| WP_027414810.1 | GT28 | NO |
| WP_027415174.1 | GT26 | NO |
| WP_027415254.1 | GT51 | NO |
| WP_027415274.1 | GH1 | NO |
| WP_027415450.1 | CE4 | SP(Sec/SPI) |
| WP_027415453.1 | AA6 | NO |
| WP_027415559.1 | CE4 | NO |
| WP_027415573.1 | GT4 | NO |
| WP_027415574.1 | GT2 | NO |
| WP_027415656.1 | CE14 | NO |
| WP_027415683.1 | GH13 | NO |
| WP_027415684.1 | GT35 | NO |
| WP_027415685.1 | GT5 | NO |
| WP_027415687.1 | CBM48 | NO |
|  | GH13 |  |
| WP_027415871.1 | GT28 | NO |
| WP_027415943.1 | GT83 | NO |
| WP_027415944.1 | GT2 | NO |
| WP_027415981.1 | CE10 | NO |
| WP_027416098.1 | CE4 | NO |
| WP_027416755.1 | AA4 | NO |
| WP_027417072.1 | GH13 | NO |
| WP_027417074.1 | GH32 | NO |
| WP_027417088.1 | GH13 | NO |
| WP_027417097.1 | GT4 | NO |
| WP_027417100.1 | GH1 | NO |
| WP_027417184.1 | GH13 | NO |
| WP_027417195.1 | CE14 | NO |
| WP_027417198.1 | CBM50 | NO |
| WP_027417229.1 | GT84 | NO |
|  | GH94 |  |
| WP_027417273.1 | GH109 | NO |
| WP_027417327.1 | GT2 | NO |
| WP_027417417.1 | CE4 | SP(Sec/SPI) |
| WP_027417519.1 | GT4 | NO |
| WP_027417618.1 | GH109 | NO |
| WP_027417721.1 | CE1 | NO |
| WP_027417910.1 | GH18 | SP(Sec/SPI) |
| WP_035100084.1 | CE4 | SP(Sec/SPI) |
| WP_035100703.1 | CE4 | NO |
| WP_035101099.1 | GT28 | NO |
| WP_035102034.1 | CE14 | NO |
| WP_047150302.1 | CE4 | SP(Sec/SPI) |
| WP_047150310.1 | GT2 | NO |
| WP_047150553.1 | GT2 | NO |
| WP_047150554.1 | GT4 | NO |
| WP_047150739.1 | GT51 | NO |
| WP_047150763.1 | SLH | SP(Sec/SPI) |
| WP_047150864.1 | GT2 | NO |
| WP_047150872.1 | GT2 | NO |
| WP_047150873.1 | GT4 | NO |
| WP_047151091.1 | GT35 | NO |
| WP_047151114.1 | GH13 | NO |
| WP_047151145.1 | GH13 | NO |
| WP_047151207.1 | CBM48 | NO |
|  | GH13 |  |
| WP_047151208.1 | GT5 | NO |
| WP_047151300.1 | AA3 | SP(Sec/SPI) |
| WP_047151326.1 | GT2 | NO |
| WP_047151346.1 | CE1 | NO |
| WP_047151553.1 | GT28 | NO |
| WP_047151902.1 | GH109 | NO |
| WP_047151939.1 | GT2 | NO |
| WP_047151971.1 | GH2 | NO |
| WP_047151975.1 | GH109 | NO |
| WP_047151983.1 | GT4 | SP(Sec/SPI) |
| WP_047152079.1 | CE4 | NO |
| WP_047152211.1 | AA4 | NO |
| WP_047152345.1 | AA3 | NO |
| WP_047152396.1 | CE4 | NO |
|  | GH18 |  |
|  | GT2 |  |
| WP_047152521.1 | AA4 | NO |
| WP_047152598.1 | GT4 | NO |
| WP_047152599.1 | CE14 | NO |
| WP_047152666.1 | GH18 | NO |
| WP_047152668.1 | GH109 | NO |
| WP_047152673.1 | GH109 | NO |
| WP_047152678.1 | GH109 | NO |
| WP_047152773.1 | GH18 | NO |
| WP_047152933.1 | GH18 | NO |
| WP_047152934.1 | GT28 | NO |
| WP_047152935.1 | GT28 | NO |
| WP_047153097.1 | AA6 | NO |
| WP_047153148.1 | GH23 | NO |
| WP_047153254.1 | CE4 | SP(Sec/SPI) |
| WP_047153450.1 | GT28 | NO |
| WP_047153490.1 | CE4 | SP(Sec/SPI) |
| WP_047153579.1 | CE4 | SP(Sec/SPI) |
| WP_047153832.1 | GT1 | NO |
| WP_047153871.1 | GT4 | NO |
| WP_047153885.1 | CE14 | NO |
| WP_047153955.1 | CE9 | NO |
| WP_047154000.1 | GT4 | NO |
| WP_047154140.1 | AA4 | NO |
| WP_047154249.1 | GH23 | NO |
| WP_047154371.1 | GT4 | NO |
| WP_047154641.1 | CE4 | SP(Sec/SPI) |
| WP_047154696.1 | AA4 | NO |
| WP_047154736.1 | AA3 | NO |
| WP_047154930.1 | GH31 | NO |
| WP_047154942.1 | CE1 | NO |
| WP_047155008.1 | CE4 | NO |
| WP_047155018.1 | AA6 | NO |
| WP_047155152.1 | CE4 | NO |
| WP_047155157.1 | CE4 | NO |
| WP_047155241.1 | GT28 | NO |
| WP_051330781.1 | GT51 | LIPO(Sec/SPII) |
| WP_051330847.1 | GT51 | NO |
| WP_051330859.1 | GT2 | NO |
| WP_051330860.1 | GT2 | NO |
|  | GT2 |  |
| WP_051330863.1 | GT4 | NO |
| WP_051331014.1 | GT2 | NO |
| WP_051331032.1 | CE4 | SP(Sec/SPI) |
| WP_051331111.1 | CE4 | NO |
| WP_051331142.1 | CE9 | NO |
| WP_051331160.1 | AA4 | NO |
| WP_051331232.1 | CBM50 | NO |
| WP_051331235.1 | GT28 | NO |
| WP_052947377.1 | CE4 | NO |
| WP_052947400.1 | GT26 | NO |
| WP_052947454.1 | GH18 | LIPO(Sec/SPII) |
| WP_052947608.1 | GT2 | NO |
| WP_052947611.1 | GT4 | NO |
| WP_052947754.1 | CE4 | SP(Sec/SPI) |
| WP_052947763.1 | GH73 | SP(Sec/SPI) |
| WP_052947915.1 | GT51 | LIPO(Sec/SPII) |
| WP_052947920.1 | GH73 | LIPO(Sec/SPII) |
| WP_052947960.1 | CBM50 | SP(Sec/SPI) |
|  | CBM50 |  |
| WP_052947997.1 | CE4 | SP(Sec/SPI) |
| WP_052948002.1 | GH23 | NO |
| WP_052948007.1 | GT51 | NO |
| WP_052948035.1 | CE4 | NO |
| WP_057897319.1 | CE4 | NO |
| WP_057897349.1 | GT51 | NO |
| WP_057897437.1 | GH109 | NO |
| WP_057897455.1 | GT26 | NO |
| WP_057897471.1 | GT4 | NO |
| WP_057897649.1 | AA4 | NO |
| WP_057898000.1 | CE4 | SP(Sec/SPI) |
| WP_057898024.1 | CE1 | NO |
| WP_057898191.1 | GT2 | NO |
| WP_057898692.1 | GT4 | NO |
| WP_057898694.1 | GH73 | SP(Sec/SPI) |
| WP_057898707.1 | CE1 | NO |
| WP_057898772.1 | GT2 | NO |
| WP_057898964.1 | CE14 | NO |
| WP_057898988.1 | GT2 | NO |
| WP_057899016.1 | GT94 | NO |
| WP_057899022.1 | CBM50 | NO |
| WP_057899271.1 | GT4 | NO |
| WP_057899459.1 | CE4 | NO |
| WP_057899607.1 | GT51 | LIPO(Sec/SPII) |
| WP_057899684.1 | GH23 | NO |
| WP_057899918.1 | AA4 | NO |
| WP_057899993.1 | GT2 | NO |
| WP_057900113.1 | CE4 | SP(Sec/SPI) |
| WP_057900303.1 | GT4 | NO |
| WP_057900380.1 | GT2 | NO |
| WP_062731155.1 | GT2 | NO |
| WP_081413182.1 | CE4 | NO |
| WP_081413217.1 | CE4 | LIPO(Sec/SPII) |
| WP_081413256.1 | PL17 | NO |
| WP_081413313.1 | CE4 | SP(Sec/SPI) |
| WP_081413373.1 | CE4 | NO |
| WP_081413401.1 | GT28 | NO |
| WP_081413449.1 | CE4 | SP(Sec/SPI) |
| WP_081413482.1 | CE4 | LIPO(Sec/SPII) |
| WP_081413503.1 | GH23 | NO |
| WP_081413527.1 | CE4 | SP(Sec/SPI) |
| WP_081413529.1 | GH18 | NO |
| WP_081413542.1 | GT4 | NO |
| WP_081413564.1 | CBM54 | SP(Sec/SPI) |
|  | SLH |  |
|  | SLH |  |
| WP_082129738.1 | GH1 | NO |
| WP_082129752.1 | GH18 | NO |
| WP_082129813.1 | GH73 | SP(Sec/SPI) |
| WP_082129826.1 | CE9 | NO |
| WP_082129856.1 | GT2 | NO |
| WP_082129862.1 | GH25 | SP(Sec/SPI) |
| WP_082129940.1 | CE4 | NO |
| WP_082129945.1 | AA6 | NO |
| WP_082129950.1 | GT51 | NO |
| WP_082130139.1 | CE4 | NO |
| WP_082130140.1 | GH25 | NO |
| WP_082130141.1 | GH8 | SP(Sec/SPI) |
| WP_082130152.1 | GT2 | NO |
| WP_082705879.1 | GT4 | NO |
| WP_082706011.1 | GH18 | NO |
| WP_082706044.1 | CE14 | NO |
| WP_082706053.1 | CE9 | NO |
| WP_082706068.1 | GT51 | NO |
| WP_091259582.1 | GT28 | NO |
| WP_091259611.1 | SLH | SP(Sec/SPI) |
| WP_091259643.1 | CE4 | SP(Sec/SPI) |
| WP_091260001.1 | GH23 | NO |
| WP_091260240.1 | AA4 | NO |
| WP_091260305.1 | GT4 | NO |
| WP_091260440.1 | GT4 | NO |
| WP_091260487.1 | AA4 | NO |
| WP_091260591.1 | GT28 | NO |
| WP_091260614.1 | GT2 | NO |
| WP_091260664.1 | GT28 | NO |
| WP_091260687.1 | CE4 | SP(Sec/SPI) |
| WP_091260755.1 | AA3 | NO |
| WP_091261016.1 | GT4 | NO |
| WP_091261018.1 | GT4 | NO |
| WP_091261025.1 | GT2 | NO |
| WP_091261190.1 | CE4 | SP(Sec/SPI) |

**Table S6: Complete list of heavy metal resistance genes found in 9 *Aneurinibacillus* strains*.***

| **Gene** | ***A. aneurinilyticus*** | ***A. danicus*** | ***A. migulanus*** | ***A. soli*** | ***Aneurinibacillus* sp. UBA3580** | ***Aneurinibacillu* sp. XH2** | ***A. terranovensis*** | ***A. thermoaerophilus*** | ***A. tyrosinisolvens*** | **Description** |
| --- | --- | --- | --- | --- | --- | --- | --- | --- | --- | --- |
| zraR/hydH | 21 | 16 | 20 | 6 | 9 | 8 | 14 | 8 | 14 | Zinc (Zn) |
| corR | 8 | 12 | 16 | 2 | 9 | 8 | 12 | 6 | 10 | Copper (Cu) |
| copR | 5 | 5 | 5 | 5 | 4 | 2 | 7 | 2 | 4 | Copper (Cu) |
| terD | 0 | 0 | 0 | 0 | 0 | 0 | 4 | 0 | 0 | Tellurium (Te) |
| mdeA | 4 | 5 | 5 | 4 | 5 | 2 | 2 | 2 | 3 | Cetrimide (CTM) [class: Quaternary Ammonium Compounds (QACs)], Benzylkonium Chloride (BAC) [class: Quaternary Ammonium Compounds (QACs)], Hoechst 33342 [class: Bisbenzimide], Ethidium Bromide [class: Phenanthridine], Rhodamine 6G [class: Xanthene], Acriflavine [class: Acridine], Tetraphenylphosphonium (TPP) [class: Quaternary Ammonium Compounds (QACs)], Chlorhexidine [class: Biguanides], Crystal Violet [class: Triarylmethane], Dequalinium [class: Quaternary Ammonium Compounds (QACs)], Pentamidine [class: Diamidine], Pyronin Y [class: Xanthene] |
| chtR | 5 | 0 | 8 | 1 | 0 | 1 | 0 | 1 | 0 | Chlorhexidine [class: Biguanides] |
| fecD | 2 | 3 | 5 | 0 | 2 | 3 | 0 | 3 | 0 | Nickel (Ni), Cobalt (Co) |
| merE | 3 | 3 | 2 | 5 | 1 | 0 | 5 | 0 | 2 | Mercury (Hg) |
| mntR | 0 | 0 | 0 | 2 | 0 | 0 | 0 | 0 | 4 | Manganese (Mn), Magnesium (Mg) |
| ruvB | 3 | 3 | 3 | 3 | 3 | 3 | 3 | 3 | 3 | Chromium (Cr), Tellurium (Te), Selenium (Se) |
| fecE | 5 | 4 | 4 | 1 | 2 | 3 | 1 | 3 | 1 | Nickel (Ni), Cobalt (Co) |
| nikD | 2 | 0 | 4 | 2 | 0 | 0 | 2 | 0 | 0 | Nickel (Ni) |
| baeR | 2 | 1 | 3 | 4 | 1 | 2 | 4 | 2 | 2 | Zinc (Zn), Tungsten (W), Sodium Deoxycholate (SDC) [class: Acid] |
| cpxR | 2 | 2 | 4 | 3 | 2 | 2 | 2 | 2 | 2 | Hydrogen Peroxide (H2O2) [class: Peroxides], Benzylkonium Chloride (BAC) [class: Quaternary Ammonium Compounds (QACs)], Chlorhexidine [class: Biguanides] |
| sodA | 3 | 2 | 2 | 3 | 1 | 2 | 2 | 3 | 1 | Selenium (Se), Hydrogen Peroxide (H2O2) [class: Peroxides] |
| acn | 2 | 2 | 2 | 2 | 2 | 2 | 2 | 2 | 2 | Iron (Fe) |
| arsT | 2 | 2 | 2 | 2 | 2 | 2 | 2 | 2 | 2 | Arsenic (As) |
| irlR | 2 | 0 | 0 | 0 | 0 | 0 | 3 | 0 | 1 | Cadmium (Cd), Zinc (Zn) |
| lmrS | 2 | 0 | 2 | 0 | 0 | 0 | 1 | 0 | 3 | Tetraphenylphosphonium (TPP) [class: Quaternary Ammonium Compounds (QACs)], Sodium Dodecyl Sulfate (SDS) [class: Organo-sulfate], Ethidium Bromide [class: Phenanthridine], Cetrimide (CTM) [class: Quaternary Ammonium Compounds (QACs)] |
| vcaM | 2 | 0 | 0 | 0 | 0 | 0 | 0 | 0 | 0 | Ethidium Bromide [class: Phenanthridine], Rhodamine 6G [class: Xanthene], 4,6-diamidino-2-phenylindole (DAPI) [class: Diamidine], Tetraphenylphosphonium (TPP) [class: Quaternary Ammonium Compounds (QACs)], Acridine Orange [class: Acridine] |
| nikE | 3 | 2 | 2 | 1 | 2 | 1 | 3 | 1 | 2 | Nickel (Ni) |
| wtpC | 1 | 3 | 1 | 1 | 3 | 1 | 2 | 1 | 0 | Tungsten (W), Molybdenum (Mo) |
| ykkC | 2 | 0 | 2 | 2 | 0 | 1 | 0 | 1 | 0 | Ethidium Bromide [class: Phenanthridine], Crystal Violet [class: Triarylmethane], Methyl Viologen [class: Paraquat] |
| ykkD | 2 | 0 | 2 | 2 | 0 | 1 | 0 | 1 | 0 | Ethidium Bromide [class: Phenanthridine], Crystal Violet [class: Triarylmethane], Methyl Viologen [class: Paraquat] |
| actP | 2 | 2 | 3 | 1 | 1 | 1 | 0 | 1 | 1 | Copper (Cu), Sodium acetate [class: Acetate] |
| blt | 1 | 0 | 2 | 0 | 0 | 0 | 0 | 0 | 0 | Ethidium Bromide [class: Phenanthridine], Rhodamine 6G [class: Xanthene], Acridine Orange [class: Acridine], Tetraphenylphosphonium (TPP) [class: Quaternary Ammonium Compounds (QACs)] |
| cadC | 1 | 2 | 1 | 2 | 2 | 0 | 0 | 0 | 1 | Cadmium (Cd), Bismuth (Bi), Zinc (Zn), Lead (Pb) |
| troB | 2 | 2 | 2 | 1 | 1 | 1 | 0 | 1 | 1 | Zinc (Zn), Manganese (Mn), Iron (Fe) |
| fabL/ygaA | 1 | 2 | 2 | 1 | 1 | 1 | 2 | 1 | 1 | Triclosan [class: Phenolic compounds] |
| glpF | 1 | 1 | 1 | 1 | 1 | 2 | 1 | 2 | 2 | Antimony (Sb), Arsenic (As), Glycerol [class: Alcohol] |
| nikB | 2 | 1 | 2 | 1 | 1 | 0 | 1 | 0 | 0 | Nickel (Ni) |
| wtpA | 0 | 1 | 1 | 0 | 0 | 0 | 2 | 0 | 0 | Tungsten (W), Molybdenum (Mo) |
| nrsS | 1 | 1 | 0 | 2 | 0 | 1 | 1 | 1 | 2 | Nickel (Ni) |
| galE | 0 | 0 | 1 | 0 | 1 | 0 | 1 | 0 | 2 | Cetyltrimethylammonium bromide (CTAB) [class: Quaternary Ammonium Compounds (QACs)] |
| mntH/yfeP | 1 | 0 | 0 | 1 | 0 | 0 | 2 | 0 | 1 | Manganese (Mn), Iron (Fe), Cadmium (Cd), Cobalt (Co), Zinc (Zn) |
| pbrA | 0 | 1 | 0 | 2 | 1 | 0 | 0 | 0 | 1 | Lead (Pb) |
| modB | 1 | 1 | 1 | 1 | 1 | 1 | 2 | 1 | 2 | Tungsten (W), Molybdenum (Mo) |
| modC | 1 | 1 | 1 | 1 | 1 | 1 | 2 | 1 | 2 | Tungsten (W), Molybdenum (Mo) |
| pstB | 1 | 1 | 1 | 1 | 1 | 1 | 2 | 1 | 2 | Arsenic (As) |
| arsM | 2 | 1 | 0 | 1 | 1 | 0 | 1 | 0 | 0 | Arsenic (As) |
| modA | 1 | 1 | 0 | 1 | 1 | 0 | 2 | 0 | 1 | Tungsten (W), Molybdenum (Mo) |
| fbpC | 2 | 1 | 1 | 1 | 0 | 1 | 0 | 1 | 1 | Iron (Fe), Gallium (Ga) |
| nikC | 1 | 1 | 2 | 1 | 1 | 0 | 1 | 0 | 1 | Nickel (Ni) |
| copA | 1 | 1 | 1 | 1 | 1 | 1 | 1 | 1 | 2 | Copper (Cu), Silver (Ag) |
| perR | 1 | 2 | 1 | 1 | 1 | 1 | 1 | 1 | 1 | Hydrogen Peroxide (H2O2) [class: Peroxides] |
| acr3 | 0 | 0 | 0 | 0 | 0 | 1 | 0 | 1 | 0 | Arsenic (As) |
| ALU1-P | 1 | 0 | 1 | 1 | 0 | 0 | 1 | 0 | 1 | Aluminium (Al) |
| arrB | 0 | 0 | 0 | 0 | 0 | 0 | 1 | 0 | 0 | Arsenic (As) |
| arsB | 0 | 1 | 1 | 1 | 0 | 0 | 1 | 0 | 1 | Arsenic (As), Antimony (Sb) |
| arsC | 0 | 1 | 1 | 1 | 0 | 1 | 1 | 1 | 1 | Arsenic (As) |
| arsR | 0 | 0 | 0 | 0 | 0 | 1 | 0 | 1 | 1 | Arsenic (As) |
| bcr | 0 | 0 | 0 | 0 | 0 | 0 | 1 | 0 | 0 | Acriflavine [class: Acridine] |
| bcrC | 0 | 1 | 0 | 0 | 1 | 0 | 0 | 0 | 0 | Benzylkonium Chloride (BAC) [class: Quaternary Ammonium Compounds (QACs)] |
| chrF | 0 | 0 | 0 | 0 | 0 | 0 | 1 | 0 | 0 | Chromium (Cr) |
| chrR | 0 | 0 | 0 | 0 | 1 | 1 | 0 | 1 | 0 | Chromium (Cr), Iron (Fe), Hydrogen Peroxide (H2O2) [class: Peroxides], 2,6-dichloroindophenol [class: Phenolic Compounds] |
| chtS | 1 | 0 | 1 | 1 | 0 | 0 | 0 | 0 | 0 | Chlorhexidine [class: Biguanides] |
| corT/coaT | 1 | 1 | 1 | 0 | 1 | 1 | 0 | 1 | 0 | Cobalt (Co) |
| ctpV | 0 | 1 | 0 | 0 | 0 | 1 | 1 | 1 | 0 | Copper (Cu) |
| cueR | 0 | 1 | 0 | 0 | 0 | 0 | 0 | 0 | 1 | Copper (Cu) |
| cusR/ylcA | 0 | 0 | 0 | 1 | 0 | 0 | 0 | 0 | 0 | Copper (Cu), Silver (Ag) |
| cutA | 1 | 1 | 1 | 0 | 0 | 0 | 0 | 0 | 0 | Copper (Cu) |
| dpsA | 1 | 1 | 1 | 0 | 1 | 0 | 0 | 0 | 0 | Iron (Fe), Hydrogen Peroxide (H2O2) [class: Peroxides] |
| emrAsm | 0 | 0 | 1 | 0 | 0 | 0 | 0 | 0 | 0 | Carbonyl cyanide 3-chlorophenylhydrazone (CCCP) [class: Hydrazone], Tetrachlorosalicylanilide (TCS) [class: Salicylanilide] |
| fabI | 0 | 0 | 0 | 1 | 0 | 0 | 0 | 0 | 0 | Triclosan [class: Phenolic compounds] |
| fabK | 0 | 0 | 0 | 0 | 1 | 0 | 1 | 0 | 1 | Triclosan [class: Phenolic compounds] |
| fbpA | 1 | 0 | 0 | 0 | 0 | 0 | 0 | 0 | 0 | Iron (Fe), Gallium (Ga) |
| fbpB | 1 | 0 | 0 | 0 | 0 | 0 | 0 | 0 | 0 | Iron (Fe), Gallium (Ga) |
| fetA/ybbL | 1 | 1 | 0 | 0 | 1 | 0 | 0 | 1 | 0 | Iron (Fe), Hydrogen Peroxide (H2O2) [class: Peroxides] |
| fetB/ybbM | 1 | 1 | 1 | 1 | 1 | 1 | 1 | 1 | 1 | Iron (Fe), Hydrogen Peroxide (H2O2) [class: Peroxides] |
| G2alt | 0 | 1 | 0 | 0 | 1 | 1 | 0 | 1 | 0 | Aluminium (Al) |
| mdrL/yfmO | 0 | 1 | 0 | 1 | 1 | 1 | 0 | 1 | 0 | Zinc (Zn), Cobalt (Cobalt), Chromium (Cr), Ethidium Bromide [class: Phenanthridine], Benzylkonium Chloride (BAC) [class: Quaternary Ammonium Compounds (QACs)] |
| mdtG/yceE | 0 | 1 | 0 | 0 | 1 | 0 | 0 | 0 | 0 | Sodium Deoxycholate (SDC) [class: Acid] |
| merA | 0 | 0 | 0 | 0 | 0 | 0 | 1 | 0 | 0 | Mercury (Hg), Phenylmercury Acetate [class: Organo-mercury] |
| merR | 0 | 0 | 0 | 0 | 0 | 1 | 0 | 1 | 0 | Mercury (Hg) |
| merR1 | 0 | 0 | 0 | 0 | 0 | 0 | 1 | 0 | 1 | Mercury (Hg) |
| mgtA | 1 | 0 | 0 | 1 | 0 | 0 | 0 | 0 | 0 | Cobalt (Co), Magnesium (Mg) |
| mmR | 0 | 0 | 0 | 0 | 0 | 1 | 0 | 1 | 1 | Tetraphenylphosphonium (TPP) [class: Quaternary Ammonium Compounds (QACs)], Acriflavine [class: Acridine], Ethidium Bromide [class: Phenanthridine], Safranin O [class: Azin], Pyronin Y [class: Xanthene] |
| mntA/ytgA | 1 | 1 | 1 | 0 | 0 | 0 | 0 | 0 | 0 | Manganese (Mn), Cadmium (Cd) |
| mtrD | 1 | 1 | 1 | 1 | 1 | 1 | 1 | 1 | 1 | Triton X-100 [class: Polyethylene glycol] |
| nikA | 0 | 0 | 1 | 1 | 0 | 0 | 0 | 0 | 0 | Nickel (Ni) |
| nikR | 0 | 1 | 0 | 0 | 1 | 0 | 0 | 0 | 0 | Nickel (Ni) |
| nixA | 0 | 0 | 0 | 0 | 0 | 0 | 1 | 0 | 0 | Nickel (Ni) |
| nmtR | 1 | 0 | 1 | 0 | 0 | 1 | 0 | 1 | 0 | Nickel (Ni), Cadmium (Cd), Lead (Pb) |
| pdrM | 1 | 1 | 1 | 1 | 1 | 1 | 0 | 1 | 1 | Acriflavine [class: Acridine], 4,6-diamidino-2-phenylindole (DAPI) [class: Diamindine] |
| pfr | 1 | 0 | 1 | 1 | 0 | 0 | 0 | 0 | 0 | Iron (Fe), Copper (Cu), Manganese (Mn) |
| pstA | 1 | 1 | 1 | 1 | 1 | 1 | 1 | 1 | 1 | Arsenic (As) |
| pstC | 1 | 1 | 1 | 1 | 1 | 1 | 1 | 1 | 1 | Arsenic (As) |
| pstS | 1 | 1 | 1 | 1 | 1 | 1 | 1 | 1 | 1 | Arsenic (As) |
| recG | 1 | 1 | 1 | 1 | 1 | 1 | 1 | 1 | 1 | Chromium (Cr), Tellurium (Te), Selenium (Se) |
| rpoS | 1 | 1 | 1 | 1 | 1 | 1 | 1 | 1 | 1 | Hydrochloric acid (HCl) [class: Acid], Sodium hydroxide (NaOH) [class: Base] |
| sh-fabI | 1 | 1 | 1 | 1 | 1 | 0 | 1 | 0 | 1 | Triclosan [class: Phenolic compounds] |
| smdA | 1 | 0 | 1 | 0 | 0 | 0 | 1 | 0 | 0 | 4,6-diamidino-2-phenylindole (DAPI) [class: Diamidine], Hoechst 33342 [class: Bisbenzimide] |
| smdB | 1 | 0 | 0 | 0 | 0 | 0 | 1 | 0 | 0 | 4,6-diamidino-2-phenylindole (DAPI) [class: Diamidine], Hoechst 33342 [class: Bisbenzimide] |
| smrA | 0 | 0 | 0 | 1 | 0 | 0 | 0 | 0 | 1 | Ethidium Bromide [class: Phenanthridine], Rhodamine 6G [class: Xanthene], Tetraphenylphosphonium (TPP) [class: Quaternary Ammonium Compounds (QACs)], 4,6-diamidino-2-phenylindole (DAPI) [class: Diamidine] |
| sugE | 0 | 1 | 0 | 1 | 1 | 0 | 0 | 0 | 0 | Cetrimide (CTM) [class: Quaternary Ammonium Compounds (QACs)], Cetylpyridinium Chloride (CPC) [class: Quaternary Ammonium Compounds (QACs)], Tetraphenylphosphonium (TPP) [class: Quaternary Ammonium Compounds (QACs)], Benzylkonium Chloride (BAC) [class: Quaternary Ammonium Compounds (QACs)], Ethidium Bromide [class: Phenanthridine], Sodium Dodecyl Sulfate (SDS) [class: Organo-sulfate] |
| troC | 1 | 1 | 1 | 0 | 0 | 0 | 0 | 0 | 0 | Zinc (Zn), Manganese (Mn), Iron (Fe) |
| troD | 1 | 1 | 1 | 0 | 0 | 0 | 0 | 0 | 0 | Zinc (Zn), Manganese (Mn), Iron (Fe) |
| ybtQ | 0 | 0 | 1 | 0 | 0 | 0 | 0 | 0 | 0 | Iron (Fe) |
| yfmO | 1 | 0 | 1 | 0 | 0 | 0 | 0 | 0 | 1 | Copper (Cu) |
| yfmP | 0 | 0 | 0 | 0 | 0 | 0 | 0 | 0 | 1 | Copper (Cu) |
| ziaA | 1 | 0 | 1 | 0 | 0 | 1 | 0 | 1 | 0 | Zinc (Zn) |
| zitB/ybgR | 0 | 1 | 1 | 1 | 1 | 1 | 0 | 1 | 0 | Zinc (Zn) |
| **Total** | **133** | **115** | **145** | **94** | **86** | **77** | **114** | **77** | **100** |  |
